# Dual‐Wavelength Responsive Hydrogel Glue with Visible‐Light Bonding and UV‐Triggered Debonding via *Ortho*‐Nitrobenzyl Cleavage

**DOI:** 10.1002/advs.202507809

**Published:** 2025-08-04

**Authors:** Huaming Wang, Xianyan Shen, Changling Du, Xian‐You Liu, Anyu Yang, Yanni Cao, Aijie Han, Qihan Liu, Jennifer Laaser, Wei Zhang

**Affiliations:** ^1^ Department of Pharmaceutical Science University of Pittsburgh Pittsburgh PA 15261 USA; ^2^ Department of Chemistry University of Pittsburgh Pittsburgh PA 15260 USA; ^3^ Department of Mechanical Engineering University of Pittsburgh Pittsburgh PA 15261 USA

**Keywords:** decrosslinking, hydrogel, ortho‐nitrobenzyl, photo‐responsive, removable glue

## Abstract

Removable adhesives with controllable bonding and debonding capabilities are essential for biomedical devices, temporary fixation, and recyclable materials. Here, a dual‐wavelength hydrogel glue is presented that integrates visible‐light polymerization (470 nm) for strong adhesion and UV‐induced degradation (365 nm) for rapid debonding. The system leverages camphorquinone as a visible‐light photoinitiator and ortho‐nitrobenzyl poly(ethylene glycol) dimethacrylate (ONB‐PEGDMA) as a UV‐cleavable crosslinker, ensuring independent control over adhesion and detachment with orthogonal polymerization and degradation. The degradation rate of ONB‐PEGDMA increases proportionally with light intensity, providing precise control over cleavage kinetics, with first‐order rate constants of 0.155, 0.278, and 0.669 min^−1^ for 20, 50, and 100 mW cm^−^^2^, respectively. The hydrogel exhibits strong adhesion (≈200 kPa) and undergoes a fourfold reduction in adhesion strength within 90 s of 365 nm irradiation at 100 mW cm^−^^2^ under a constant tensile load of 10 N, enabling efficient removal. Rheological analysis confirms a significant decrease in storage modulus and crosslinking density after UV exposure, leading to network softening and structural failure. This work pioneers a phototunable hydrogel glue that bridges photopolymerization and photodegradation, offering a promising platform for next‐generation adhesives with precise spatiotemporal control and with easy application, good bonding, and rapid UV‐triggered debonding.

## Graphical abstract

A dual‐wavelength responsive hydrogel glue achieves orthogonal control of bonding and debonding: visible light drives crosslinking via acrylic acid, while ortho‐nitrobenzyl (ONB) units enable rapid UV‐triggered network cleavage. This smart adhesive offers strong adhesion, fast on‐demand debonding, and spatiotemporal precision for potential applications in biomedical and recyclable materials.

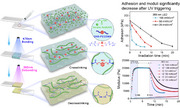

## 1 Introduction

Adhesives play a crucial role in numerous applications, spanning from industrial manufacturing and electronics assembly to biomedical devices and wound closure.^[^ ^1^ ^]^ They can be broadly categorized into various types, such as liquid adhesives (e.g., glues), pressure‐sensitive adhesives (e.g., tapes), hot‐melt adhesives, spray adhesives, and film adhesives (e.g., pre‐formed sheets or patches). For instance, glues are typically applied in a liquid state and solidify in situ, forming strong bonds, and tapes utilize surface interactions for attachment. While traditional glues, such as cyanoacrylate‐based “super glues” and epoxy resins, provide strong and durable adhesion, they are difficult to remove. On the other hand, the increasing demand for functional and adaptable materials has highlighted the need for removable adhesives that can meet dynamic requirements.^[^ ^2^ ^]^ The inability to selectively remove or debond creates challenges in repositioning, reusing parts, or biomedical settings where controlled adhesion and removal are desired. The challenges have spurred interest in stimuli‐responsive glues, which enable controlled debonding using thermal,^[^ ^3^ ^]^ light,^[^ ^4^ ^]^ electrical,^[^ ^5^ ^]^ magnetic^[^ ^6^ ^]^ or chemical triggers.^[^ ^7^ ^]^ Thermal‐debonding systems are currently the most common methods, but require elevated temperatures to achieve debonding, limiting their use in biological or temperature‐sensitive settings.^[^ ^8^ ^]^ Light‐responsive adhesives offer spatial and temporal precision as well as non‐contact activation and minimal mechanical stress on substrates. Despite these advantages, existing systems remain suboptimal. Light‐responsive glues have been reported, but still often face trade‐offs between bonding strength, responsiveness, and ease of application. Photothermal systems^[^ ^9^ ^]^ can enable rapid removal but still generate high surface temperatures. Azobenzene‐based systems,^[^ ^4^, ^10^ ^]^ while fast‐switching, often need heat‐assisted initial bonding as powdered precursors exhibit limited fluidity under UV light alone. Supramolecular systems^[^ ^11^ ^]^ typically offer relatively weak bonding. There are also other systems, including ortho‐nitrobenzyl (ONB)‐containing polymers,^[^ ^12^ ^]^ that require high UV doses and exhibit slow or incomplete cleavage. Therefore, we propose to design a hydrogel glue that achieves a practical balance of ease of application, good bonding, and rapid UV‐triggered debonding, all within a simple aqueous formulation.

Hydrogels have emerged as a promising class of materials as adhesives due to their tunable mechanical properties, flexibility, and adaptability to wet environments, etc.^[^ ^13^ ^]^ They have been widely used in drug delivery,^[^ ^14^ ^]^ wound dressing,^[^ ^15^ ^]^ tissue engineering,^[^ ^16^ ^]^ agriculture^[^ ^17^ ^]^ and many other applications. While light‐responsive hydrogels have been extensively studied, their use in photo‐degradable glues is still underdeveloped. Most photo‐triggered hydrogel glues typically use light‐induced crosslinking to form adhesion, rather than inducing rapid and efficient detachment. In addition, different from adhesive tapes, which can rely on surface property changes for triggered detachment, glues would require bulk degradation for complete removal. Achieving both strong adhesion and easy detachment has been a challenge. Developing dual‐wavelength responsive hydrogel glues that integrate photoinduced strong adhesion with fast photodegradation, which can facilitate efficient network breakdown without compromising initial adhesion, would mark a meaningful step forward in the responsive adhesive technologies.^[^ ^18^ ^]^

ONB derivatives are among the most extensively studied photocleavable functional groups due to their efficient photolysis under UV light and synthetic accessibility.^[^ ^19^ ^]^ ONB chemistry has been widely applied in the design of photo‐responsive polymers for diverse applications.^[^ ^12^, ^20^ ^]^ ONB‐containing crosslinkers have been incorporated into hydrogels to enable light‐triggered degradation and modulation of physical properties.^[^ ^21^ ^]^ Recently, ONB‐based design has also been explored for debondable adhesives by incorporating ONB moieties into polymer backbones.^[^ ^22^ ^]^

**Figure 1:**
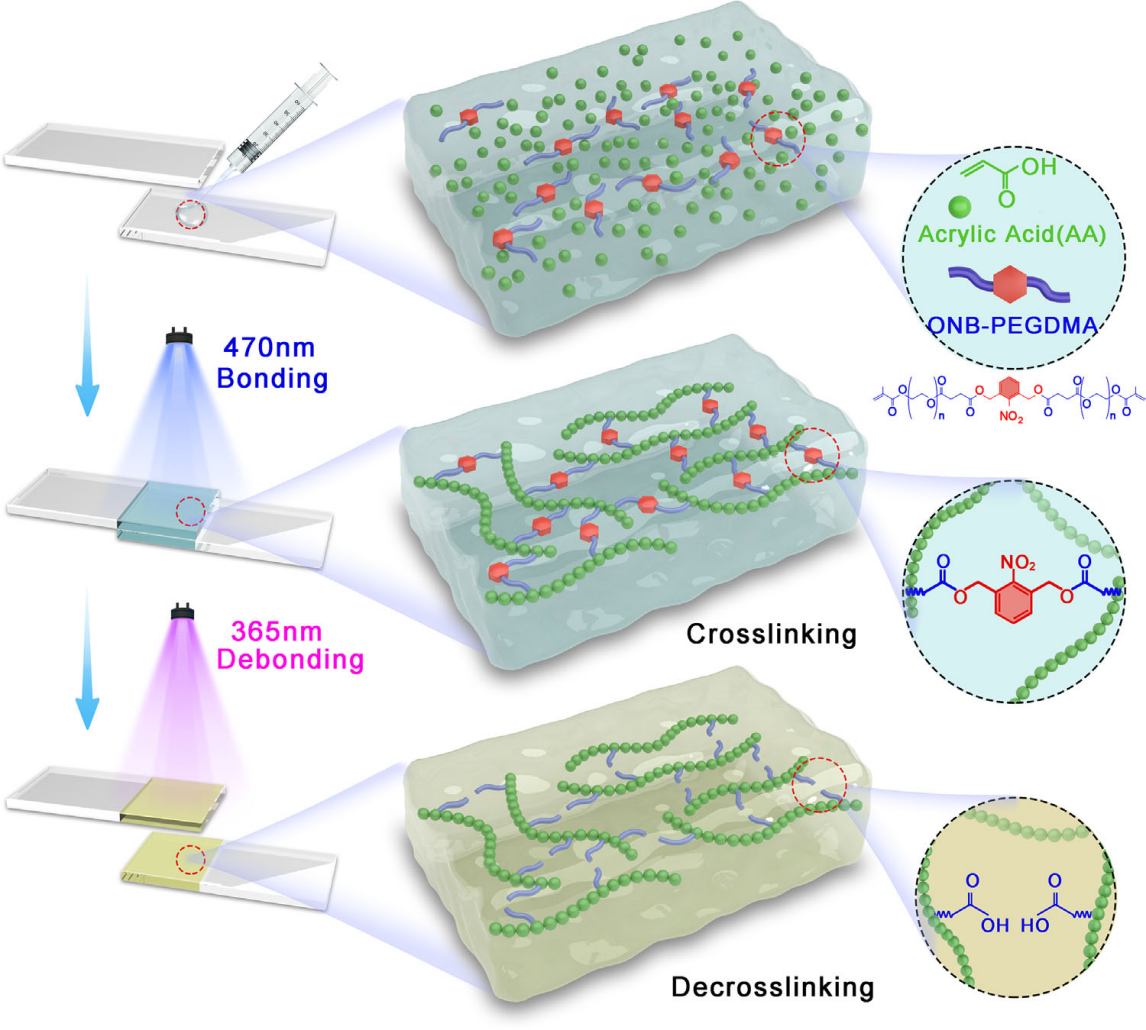
Schematic representation of dual‐wavelength responsive hydrogel glue that integrated visible‐light polymerization and UV‐induced degradation.

In this study, we present a novel dual‐wavelength responsive hydrogel glue that integrates visible‐light polymerization and UV‐induced rapid degradation. The hydrogel matrix consisted of polyacrylic acid (PAA) crosslinked with poly(ethylene glycol) (PEG) dimethacrylate containing an ONB moiety (**Figure** **1**). The system utilized camphorquinone (CQ) as a visible‐light photoinitiator and ONB moieties as UV‐sensitive depolymerization triggers to achieve control over both adhesion and detachment by different light. A challenge in dual‐responsive adhesives is ensuring no interference between photodegradation and polymerization. Therefore, we strategically designed the ONB linker with absorption at relatively short wavelengths, yet still cleavable at 365 nm, allowing polymerization and degradation to proceed orthogonally. Additionally, only a low concentration of ONB moieties was needed, ensuring efficient debonding in a short time without compromising initial adhesion.

## 2 Results and Discussion

**Figure 2:**
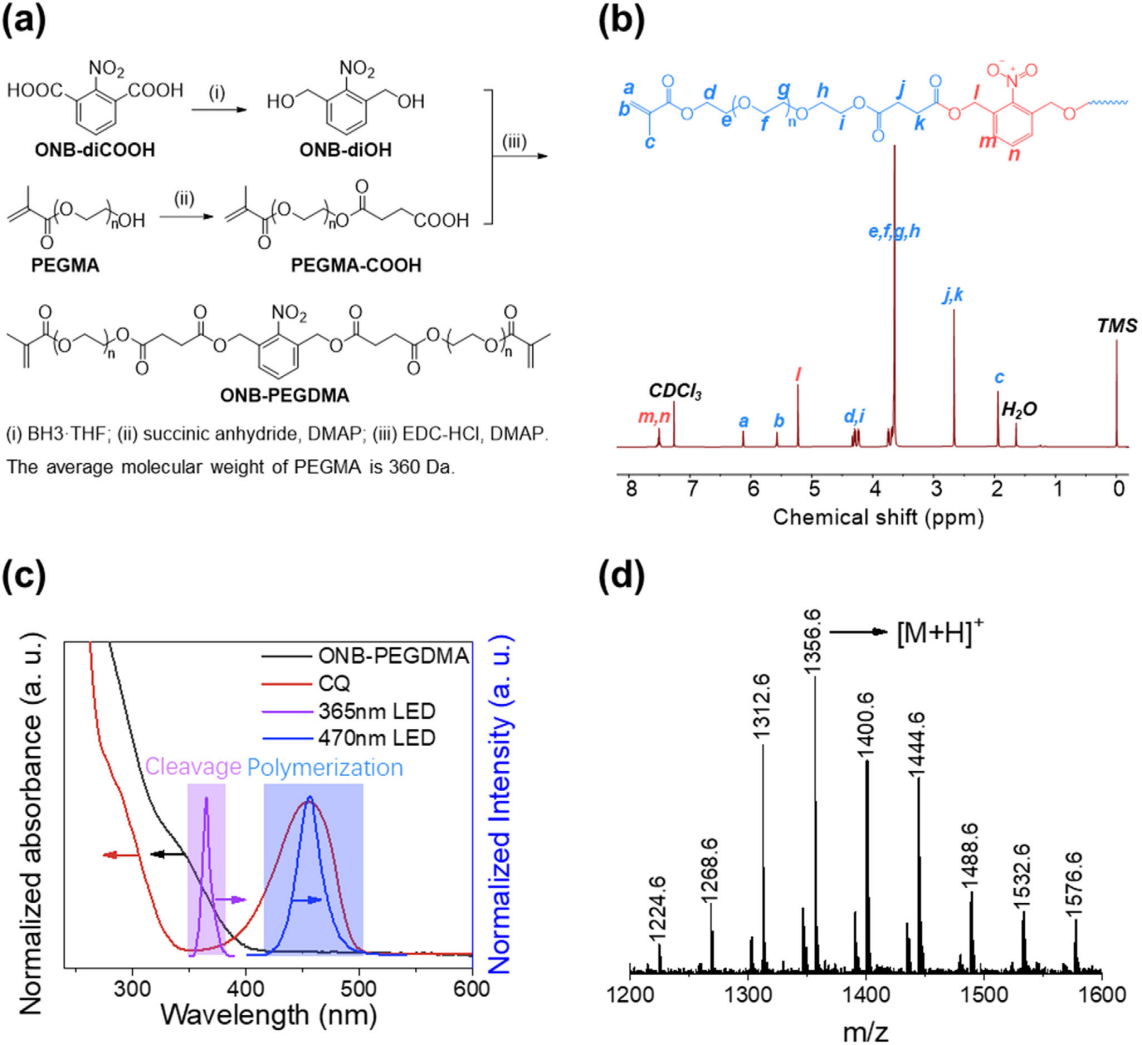
Synthesis and characterization of ONB‐PEGDMA. a) Synthesis route of ONB‐PEGDMA. b) ^1^H NMR of ONB‐PEGDMA in CDCl_3_. c) UV–vis absorption spectra of ONB‐PEGDMA, camphorquinone in water, and emission spectra (blue and purple lines) of the LEDs used. d) Electrospray ionization mass spectrum (ESI‐MS) of ONB‐PEGDMA (M = 1355.6 Da, C_62_H_101_O_31_N, containing PEG repeating units of 19).

To achieve photo‐debonding of the adhesive, we designed a photo‐cleavable linker with an ONB moiety in PEG dimethacrylate (ONB‐PEGDMA). The PEG segment was introduced to improve water solubility. The molecular weight of ONB‐PEGDMA was 1k Da. The synthetic route is illustrated in **Figure** **2**a. In brief, the carboxylic acid groups in 2‐nitroisophthalic acid were reduced to benzyl alcohols, which then reacted with a monomethacrylate‐functionalized PEG with a carboxylic group (PEGMA‐COOH) to give ONB‐PEGDMA. The successful synthesis was confirmed by nuclear magnetic resonance (NMR) spectroscopy, gel permeation chromatography (GPC), and mass spectroscopy (Figure 2b,d; Figures S1–S9, Supporting Information). The ^1^H NMR spectrum of ONB‐PEGDMA exhibited characteristic peaks, including the aromatic protons of ONB at 7.53 ppm and the benzyl methylene protons at 5.24 ppm, which were shifted from 4.55 ppm upon successful connection with PEGMA‐COOH. ONB‐PEGDMA aqueous solution showed broad absorption in the UV region, with a threshold at 394 nm and an absorption coefficient of 1 × 10^3^ L mol^−1^ at 365 nm (Figure 2c). Its absorption above 400 nm is negligible, therefore no overlap with the 470 nm blue LED light. This separation of wavelengths ensures that the photoinitiation does not interfere with the integrity of the ONB structure.

**Figure 3:**
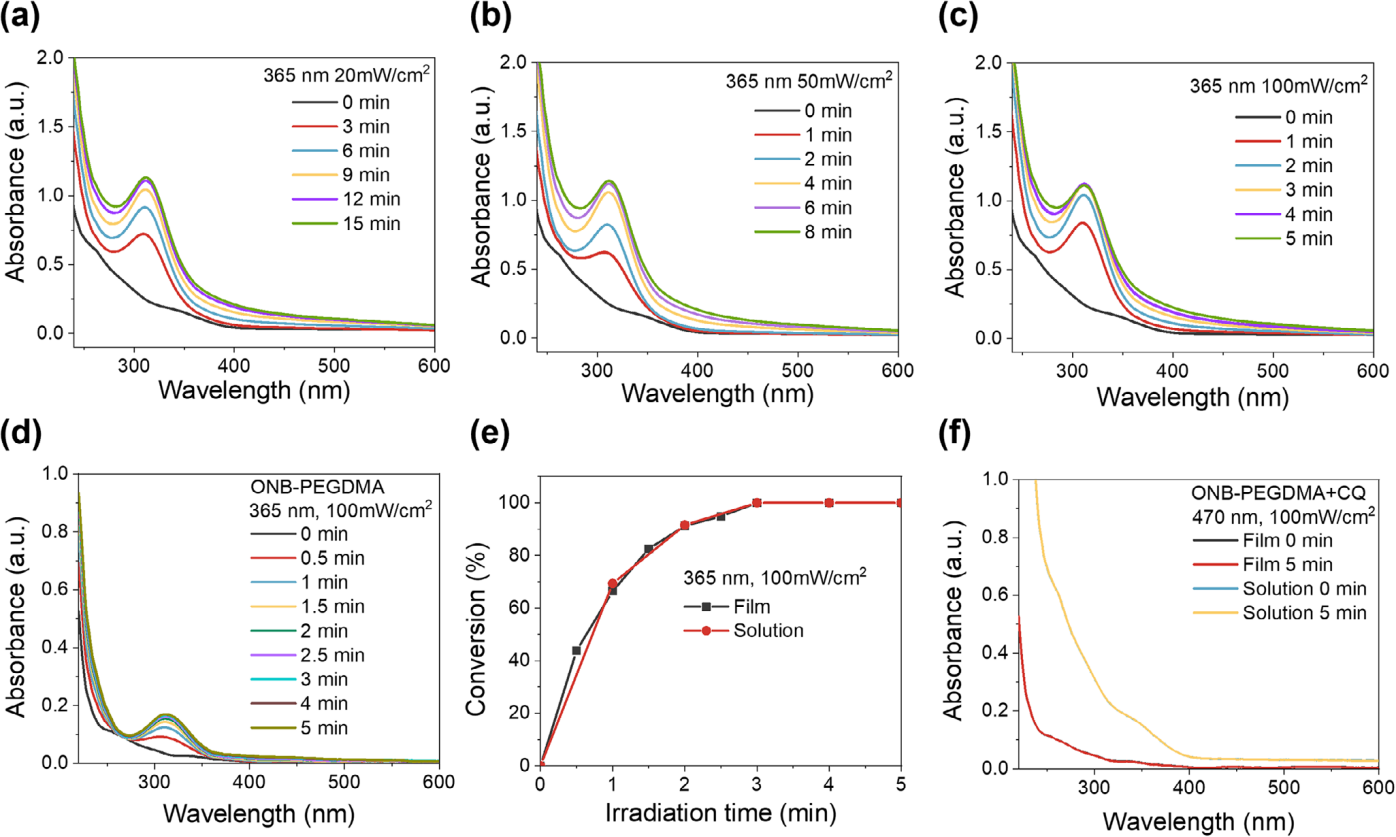
Cleavage of ONB‐PEGDMA under 365 nm irradiation a–c) UV–vis spectra of ONB‐PEGDMA aqueous solution (0.66 mg mL^−1^) under irradiation at (a) 20 mW cm^−2^, (b) 50 mW cm^−2^, and (c) 100 mW cm^−2^. d) UV–vis spectra of ONB‐PEGDMA solid film under 365 nm irradiation at 100 mW cm^−2^. e) Photocleavage conversion of ONB‐PEGDMA calculated from the peak increase at 310 nm in the UV–vis spectra. f) UV–vis spectra of ONB‐PEGDMA+CQ (20:1) aqueous solution and film under 470 nm irradiation at 100 mW cm^−2^ for 5 min, showing no spectral change.

Next, we investigated the photocleavage reaction of ONB‐PEGDMA under 365 nm LED irradiation. Upon irradiation (20, 50, or 100 mW cm^−2^), a new peak emerged at ≈310 nm (**Figure** **3**a–c), indicating the degradation of the ONB moiety.^[^ ^23^ ^]^ The intensity of this peak increased with irradiation time, rising rapidly at first and then gradually slowing as the reaction approached completion. The thin film of ONB‐PEGDMA exhibited a similar UV–vis spectral evolution as in solution upon 365 nm irradiation, with comparable photodegradation kinetics (Figure 3d,e). In addition, no change of the ONB in solution and film was confirmed after 470 nm irradiation with the presence of the photo initiator (Figure 3f; Figure S10, Supporting Information).

**Figure 4:**
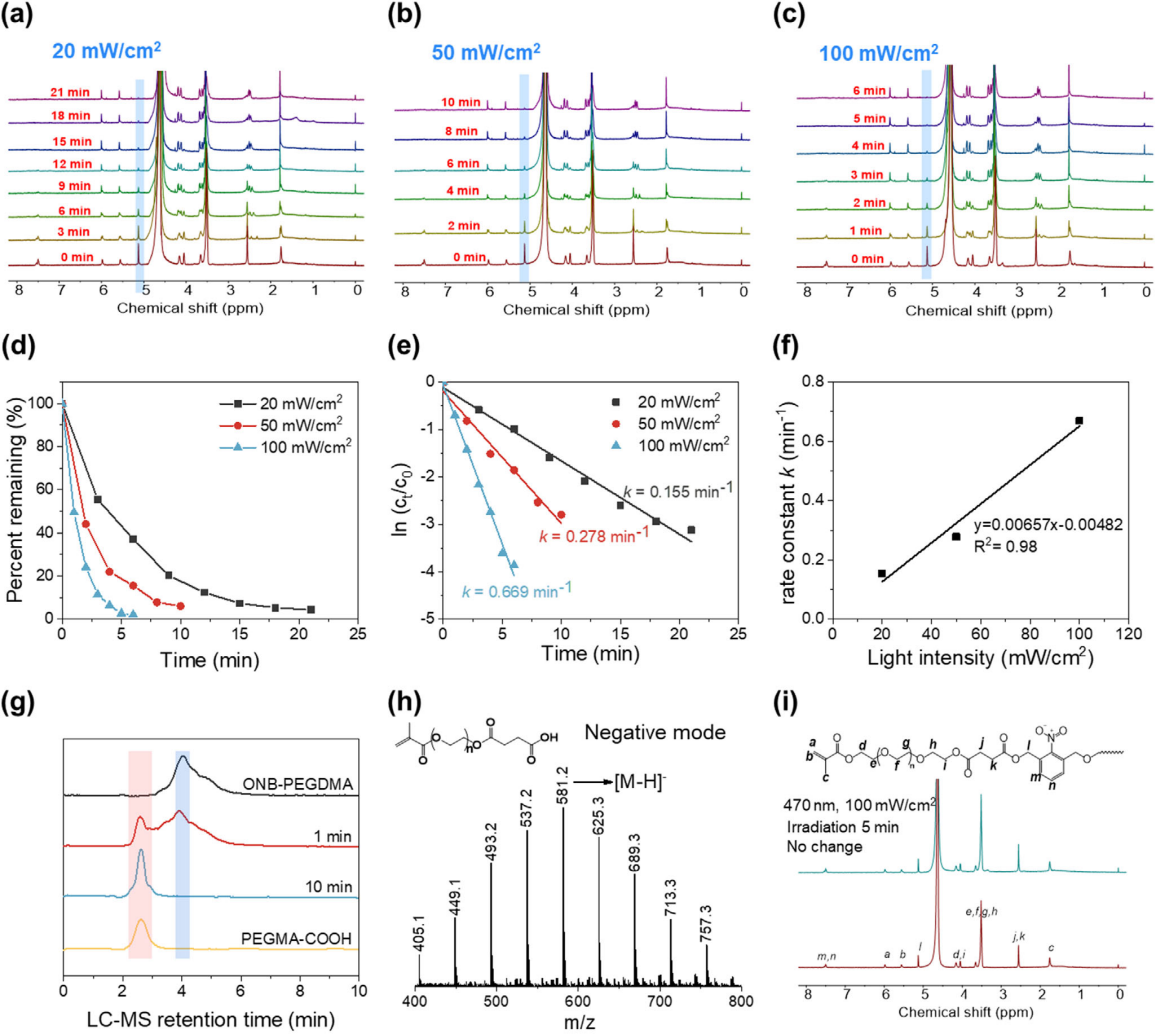
a–c) ^1^H NMR spectra of ONB‐PEGDMA in D_2_O (0.66 mg mL^−1^) under irradiation at (a) 20 mW cm^−2^, (b) 50 mW cm^−2^, and (c) 100 mW cm^−2^. d) Conversion of the photocleavage reaction from the NMR analysis under different light intensities. e) Kinetics fitting curves of ONB‐PEGDMA photocleavage. f) Linear correlation between the cleavage rate constant and light intensity. g) Liquid chromatography‐mass spectrometry (LC‐MS) curves of ONB‐PEGDMA before and after irradiation at 100 mW cm^−2^. h) MS spectrum of PEGMA‐COOH generated from 10 min of photocleavage reaction. i) ^1^H NMR spectra of ONB‐PEGDMA in D_2_O before and after irradiation under 470 nm light at 100 mW cm^−2^ for 5 min.

The cleavage kinetics were further monitored by NMR in D_2_O (**Figure** **4**a–c). Upon irradiation, the benzyl ester methylene at 5.24 ppm decreased, confirming photodegradation. The degradation followed first‐order kinetics (Figure 4d,e) with a rate constant *k* of 0.155 min^−1^ under 20 mW cm^−2^, corresponding to a half‐life (*t*_1/2_) of 4.47 min, with complete cleavage achieved in ≈20 min. Increasing light intensity accelerated the reaction while maintaining first‐order kinetics. At 50 mW cm^−^^2^, *k* increased to 0.278 min^−1^ (t₁/_2_ = 2.49 min), and at 100 mW cm^−^^2^, *k* further increased to 0.669 min^−1^ (t₁/_2_ = 1.03 min). The degradation rate increased proportionally with the light intensity (Figure 4f), revealing a direct phototriggered dependency for precise control of the cleavage. At 100 mW cm^−^^2^, complete degradation occurred within 5 min, highlighting the system's potential for rapid, controllable adhesion debonding. Besides, it was noticed that the photocleavage was significantly slower in the non‐aqueous solvent acetonitrile, but accelerated upon the addition of water. (Figure S11, Supporting Information).

For double‐substituted ONB structures, literature reports vary, with some studies indicating cleavage on one side,^[^ ^21^, ^24^ ^]^ while some observed cleavage on both sides.^[^ ^25^ ^]^ In our experiments, photocleavage occurred on both sides of the ONB. Initially, cleavage of one side generated a PEGMA‐COOH, and an o‐nitrosobenzyl intermediate retaining the remaining PEG chain was detected in liquid chromatography‐mass spectrometry (LCMS) (Figure S12, Supporting Information). This intermediate underwent further cleavage, releasing another PEGMA‐COOH molecule. NMR confirmed the release of PEGMA‐COOH (Figure S13, Supporting Information). LCMS analyses also revealed a new peak post‐cleavage, matching PEGMA‐COOH in both elution time and molecular weight (Figure 4g,h).

We subsequently incorporated ONB‐PEGDMA as a photolabile crosslinker in the polymerization of acrylic acid to form the hydrogel glues. The cure kinetics were investigated by Fourier transform infrared spectroscopy (FTIR) and rheology (Figure S14, Supporting Information). The bands at 1612 and 980 cm^−1^ correspond to the stretching vibration and bending vibration of the vinyl C═C bonds, respectively. As irradiated (470 nm, 20 mW cm^−2^), the 980 cm^−1^ peak decreased, indicating the consumption of C═C groups and formation of C─C bonds. Polymerization conversion was estimated by integration of the 980 cm^−1^ peak area, which showed ≈80% conversion in 2 min of irradiation. In parallel, the storage modulus (G’) in rheological tests also reached a plateau with the same period, confirming the completion of gelation. Both PEGDA and ONB‐PEGDMA formulations showed similar behavior, suggesting comparable reactivity. Those hydrogel glues can adhere to various substrates (glass, rubber, PTFE, paper, polypropylene, steel, polystyrene, wood, and skin) as shown in Figure S15 (Supporting Information). The adhesion originated primarily from hydrogen bonding interaction between the carboxyl groups of PAA and the substrate, whereas the crosslinker reinforced the cohesion strength of the hydrogel network.

**Figure 5:**
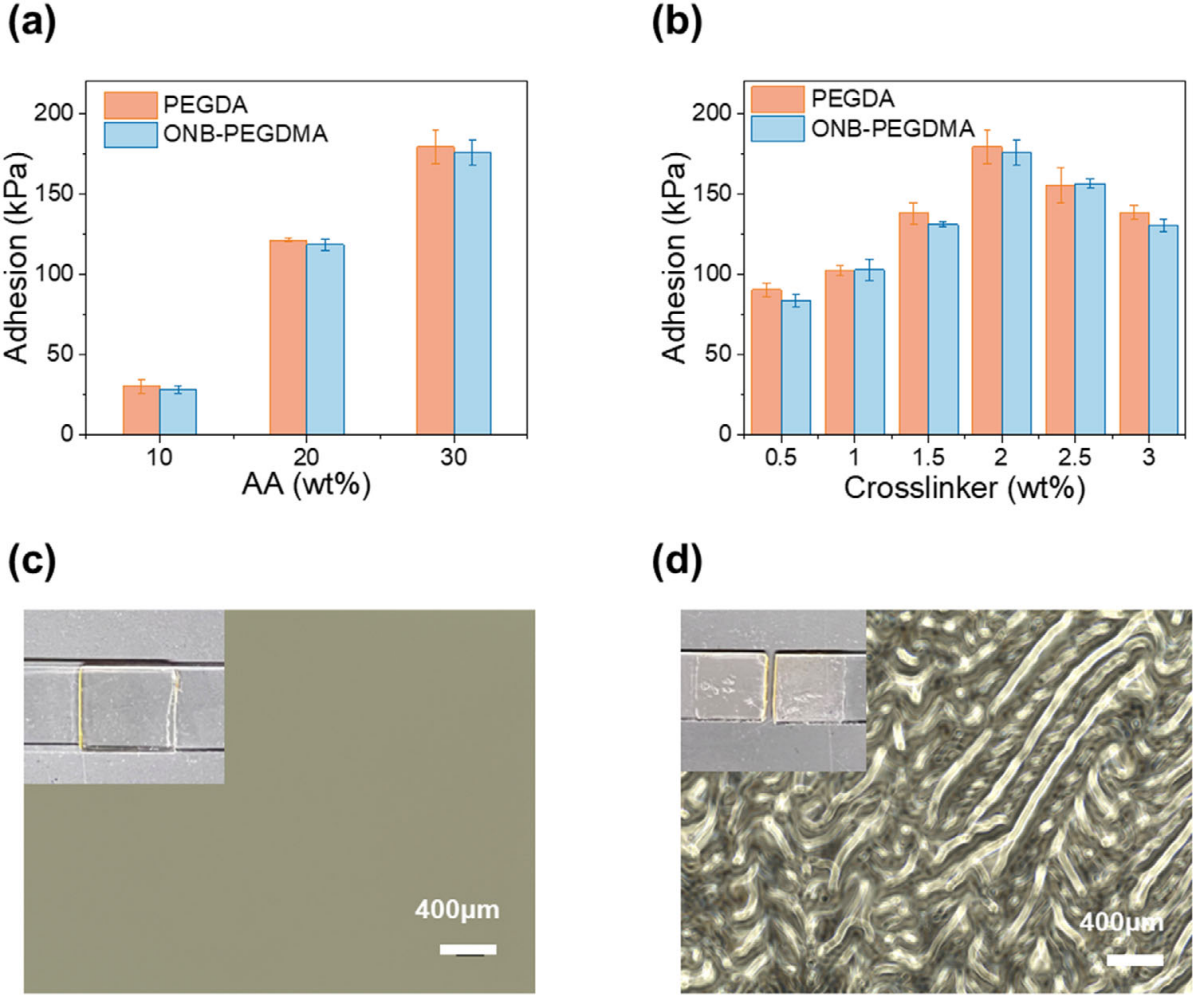
Lap shear tests of hydrogel glues composed of PAA/PEGDA and PAA/ONB‐PEGDMA. a) Adhesion strength as a function of acrylic acid concentration, b) Adhesion strength as a function of crosslinker concentration. c) Phase‐contrast microscope images of PAA30/ONB‐PEGDMA2 adhered glass slides, and d) fractured materials on glass slides.

We first studied acrylic acid (AA) concentrations (10%, 20%, 30%) on the adhesive properties (**Figure** **5**a) in a lap shear test. The glue was applied between two pieces of glass slides with an overlapping of 12 mm x 12 mm. Then, a 470 nm dental LED light (20 mW cm^−2^, 3 min) was used to trigger polymerization to form adhesion. The adhesion increased significantly (from 30.1 ± 4.4 to 179.4 ± 10.6 kPa) with AA concentrations (10–30%), attributed to denser networks that enhanced cohesive force and more binding sites for adhesive force. The comparable adhesion observed between PAA/PEGDA and PAA/ONB‐PEGDMA indicated that incorporating the ONB structure did not compromise adhesion. Next, we fixed the AA concentration at 30% and varied the PEGDA concentration (0.5%, 1%, 1.5%, 2%, 2.5%, 3%). The adhesion strength peaked at 2% PEGDA (Figure 5b). This trend of increasing adhesion followed by a decline suggests that there is an optimal crosslinker concentration for maximum bonding strength. This decline at higher crosslinker concentrations could be attributed to restricted PAA molecular movement and, therefore, limited adhesive interactions. When ONB‐PEGDMA was used as the crosslinker instead of PEGDA, the result exhibited a similar trend (Figure 5a,b) with no significant differences, confirming that PEGDA and ONB‐PEGDMA provided comparable crosslinking effects. The small ONB moiety did not alter the adhesive properties, ensuring that debonding functionality can be introduced without sacrificing bonding performance. Adhesion failure occurred within the gel (Figure 5c,d), suggesting that overall adhesion was cohesion‐controlled. This suggested the potential for achieving removability through photodegradation of the network.

**Figure 6:**
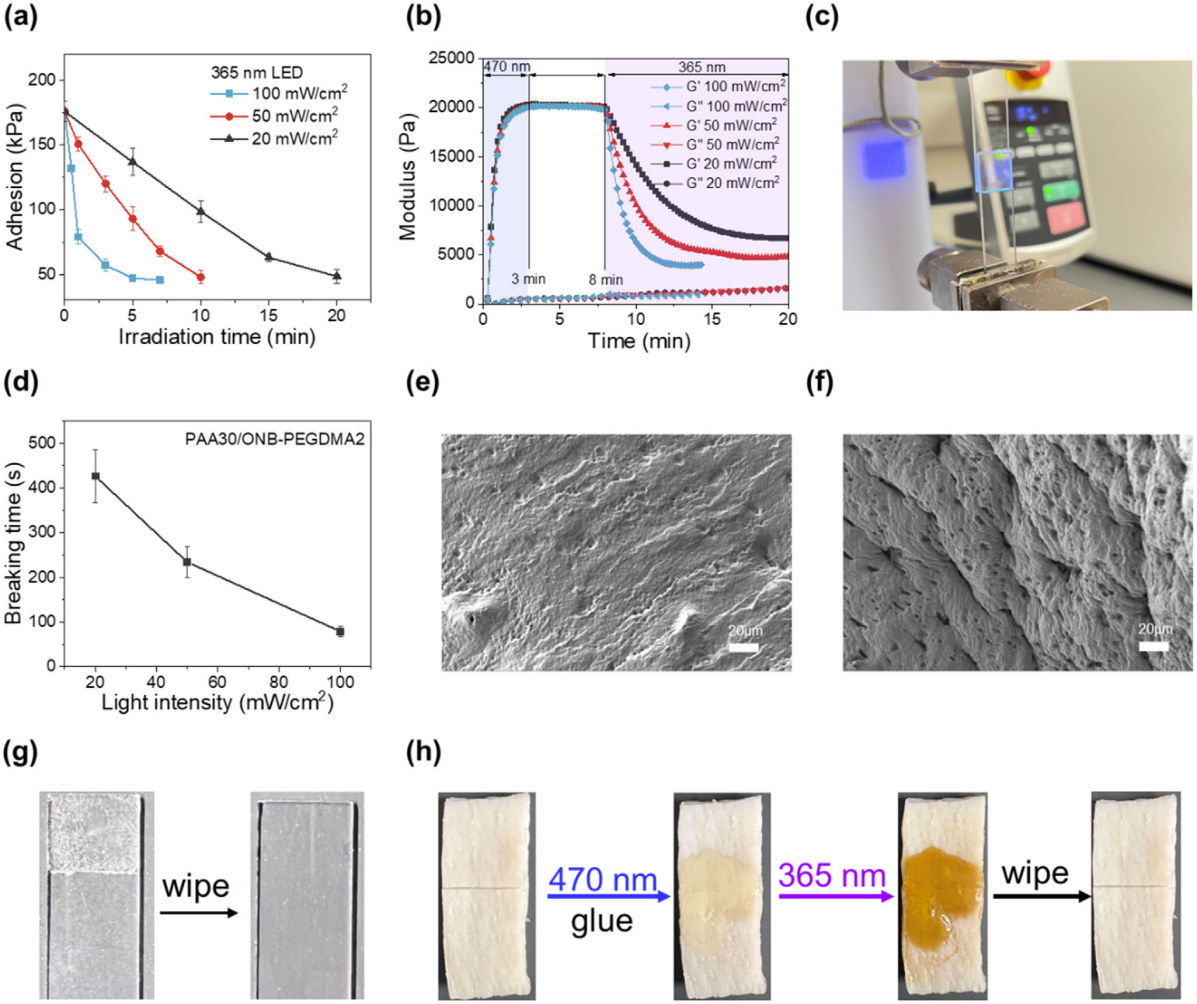
Photoinduced debonding of PAA30/ONB‐PEGDMA2 glue. a) Adhesion strength from lap shear tests after different irradiation times and intensities. b) Rheological analysis showing moduli changes during hydrogel formation under 470 nm light (20 mW cm^−2^), followed by photoinduced decrosslinking under 365 nm light (20, 50, or 100 mW cm^−2^). c) Experimental setup of debonding time measurement under a constant tensile load (10 N). d) Breaking time of adhesive under different light intensities. e) Scanning electron microscopy images of PAA30/ONB‐PEGDMA2 hydrogels before and f) after 365 nm irradiation. g) Photos of wiping adhesive residue off a glass slide. h) Photos of the removal process of adhesive (PAA30/ONB‐PEGDMA2) on porcine skin.

Next, we investigated the photoinduced debonding of the glue crosslinked with ONB‐PEGDMA. The materials were first applied between glass slides and polymerized, forming a robust adhesive layer. Then, a 365 nm LED was used to trigger network degradation for debonding. Using PAA30/ONB‐PEGDMA2 as a representative formulation, irradiation at 20 mW cm^−^^2^ for 10 min reduced adhesion strength from 175.9 ± 7.8 to 98.6 ± 8.5 kPa due to the decomposition of the ONB‐PEGDMA (**Figure** **6**a). Extending the irradiation to 20 min, the adhesion further decreased to 48.5 ± 5.4 kPa, a fourfold reduction from the initial value. As a result, the two pieces of glass slides can be easily separated, and the residue glue can be wiped off (Figure 6g).

The structural changes of the hydrogel network during photodegradation were further investigated by rheological analysis. Following 470 nm LED initiation, the adhesive network formed within 3 min, as evidenced by the plateau storage modulus (*G'* = 20.18 kPa, Figure 6b). Upon 365 nm irradiation at 20 mW cm^−^^2^, a rapid reduction in *G'* was observed, reaching a stable value (6.74 kPa) after 20 min. The overall decrease in *G'* was 66.6%, correlating with the adhesive weakening in lap shear tests. The decrease in crosslinking density (*n*_e_) was calculated using rubber elasticity theory^[^ ^26^ ^]^: $n_{e}=\frac{G^{\prime }}{RT}$, where *G’* is the storage at the plateau, *R* is the gas constant (8.314 J⋅mol^−1^⋅K^−1^ and *T* is absolute temperature (298.15 K). The calculated crosslinking density decreased from 8.14 × 10^−6^ to 2.72 × 10^−6^ mol cm^−^^3^, confirming that photodegradation effectively disrupted the cohesive integrity of the adhesive network.

As light intensity increased, the reduction of adhesion became more pronounced, aligning well with the previously observed photodegradation kinetics of ONB‐PEGDMA, where the degradation rate was proportional to light intensity. At 50 mW cm^−^^2^, adhesion reduction (to 47.9 ± 5.1 kPa) was achieved within 10 min of irradiation, accompanied by 76.6% reductions in *G’* (to 4.72 kPa) and crosslinking density (to 1.90 × 10^−6^ mol cm^−^^3^), respectively. Increasing the light intensity to 100 mW cm^−^^2^ further accelerated the process, achieving adhesion reduction (to 47.1 ± 2.1 kPa) in just 5 min, with corresponding 81.1% decreases in modulus (to 3.82 kPa) and crosslinking density (to 1.54 × 10^−6^ mol cm^−^^3^). Stress‐displacement curves showed greater displacement for samples subjected to longer irradiation (Figure S16, Supporting Information), further indicating softening of the network. Changes in microstructural morphology were characterized by scanning electron microscopy (SEM). As shown in Figure 6e,f, the irradiated hydrogels exhibited formation of numerous micropores, suggesting significant disruption of the network structure after light exposure. Besides, differential scanning calorimetry (DSC) analysis (Figure S17, Supporting Information) was conducted to assess glass transition temperature (*T*_g_) changes before and after 365 nm irradiation. The freeze‐dried sample showed a *T*_g_ of 86.5 °C before irradiation, which decreased to 80.3 °C after irradiation, indicating increased chain mobility from network decrosslinking.

Hydrogels containing 1% and 3% ONB‐PEGDMA exhibited similar trends as the 2% formulation in adhesion strength, modulus, crosslink density, and *T*_g_ before and after decrosslinking (**Table** **1**; Figures S17 and S18, Supporting Information). Increasing crosslinker content led to higher modulus and crosslink density, which both decreased by ≈80% after 365 nm irradiation. The adhesive strengths also decreased by 70% after decrosslinking. These results strengthen the correlation between crosslink density and adhesion strength. All the *T*_g_ values of the freeze‐dried PAA/ONB‐PEGDMA were lower than that of pure PAA (96.3 °C) and decreased with increasing ONB‐PEGDMA content, attributed to the flexible structure of ONB‐PEGDMA enhancing chain mobility. After UV‐triggered decrosslinking, all networks exhibited a further decreased *T*_g_ due to increased mobility.

**Table 1:** Adhesion strength, crosslink density, and glass transition temperature (*T*_g_) of PAA/ONB‐PEGDMA hydrogels before and after 365 nm irradiation (100 mW cm^‐^^2^).

Samples	Before 365 nm irradiation			After 365 nm irradiation			Reduction		
	Adhesion [kPa]	Crosslink density [× 10^−6^ mol/cm^3^]	*T*_g_ [°C]	Adhesion [kPa]	Crosslink density [× 10^−6^ mol/cm^3^]	*T*_g_ [°C]	Adhesion	Crosslink density	Δ*T*_g_ [°C]
PAA30/ONB‐PEGDMA1	102.9	6.78	90.8	32.7	1.22	83.6	68.2%	82.0%	7.2
PAA30/ONB‐PEGDMA2	175.9	8.14	86.5	47.1	1.54	80.3	73.2%	81.1%	6.2
PAA30/ONB‐PEGDMA3	130.6	9.95	78.2	40.2	2.36	65.6	69.2%	76.3%	12.6

To quantify the debonding time under load, PAA30/ONB‐PEGDMA2 hydrogel glues were subjected to a constant 10 N tensile force using a universal testing machine, followed by 365 nm light irradiation at various intensities. The experimental setup is shown in Figure 6c. As shown in Figure 6d and Figure S19 (Supporting Information), adhesive failure occurred after 426 ± 59.3 s at 20 mW cm^−^^2^, 235 ± 34.2 s at 50 mW cm^−^^2^, and just 79 ± 12.1 s at 100 mW cm^−2^. These results revealed the system's efficient, controlled, and rapid light‐induced debonding performance. The hydrogel glues were also applied to porcine skin, as shown in Figure 6h. Under 470 nm irradiation, two cut skin pieces were bonded, and the glue was easily removed after 365 nm irradiation, suggesting promising potential in biomedical applications. The dual‐wavelength responsive hydrogel glue enabled both strong visible‐light‐induced bonding and rapid UV‐triggered debonding. Robust initial adhesion is conveniently achieved through polymerization under 470 nm blue light, while the ONB groups remain unaffected. Upon exposure to 365 nm UV light, the network underwent cleavage of the ONB moieties, leading to over 80% reduction in modulus and more than 70% loss in overall adhesive strength—allowing clean and convenient removal. Another advantage of this system is its rapid response. Instead of incorporating photo‐responsive groups into the polymer backbone or side chains,^[^ ^11^, ^12^ ^]^ ONB moieties were installed at the crosslinking points, enabling efficient structural disassembly with minimal cleavage. As a result of the fast photo‐degradation kinetics and low crosslinker concentration, the system achieved debonding within a few minutes—a practical and competitive timeframe that is among the faster light‐responsive glue systems reported, with a balance of ease of application, good bonding, and rapid UV‐triggered debonding.

## 3 Conclusion

This study presents a dual‐wavelength responsive hydrogel glue that integrates visible‐light‐induced polymerization for strong adhesion with UV‐triggered degradation for rapid debonding. The wavelength selectivity of CQ and ONB enabled independent and precise control over bonding and debonding. The hydrogel exhibited high initial adhesive strength, which significantly decreased within minutes of 365 nm irradiation, enabling clean and convenient removal. The rapid cleavage of ONB‐PEGDMA was attributed to its favorable photocleavage kinetics and low concentration at the crosslinking points of the network. Rheological analysis confirmed that UV exposure markedly reduced the storage moduli and crosslinking densities, leading to network softening and adhesive failure. In contrast, hydrogels without ONB remained stable under UV, indicating that debonding is specifically triggered by ONB cleavage. By employing a simple yet robust design that coupled photopolymerization and photodegradation, this work represents a meaningful step toward practical, smart adhesives with potential applications in biomedical engineering, and temporary fixation or recyclable systems.

## 4 Experimental Section

### Materials and Reagents

The chemicals and solvents were used as received: 2‐Nitroisophthalic acid (Ambeed, 98%), (T‐4)‐trihydro(tetrahydrofuran)boron (BH_3_·THF, Thermo Scientific Chemicals, 1 m solution), Poly(ethylene glycol) methacrylate (Sigma–Aldrich, M_n_ 360), Succinic anhydride (Ambeed, 97%), 4‐Dimethylaminopyridine (Sigma–Aldrich, 99%), N‐(3‐Dimethylaminopropyl)‐N'‐ethylcarbodiimide hydrochloride (EDC‐HCl, Fisher/eMolecules, 99%), Camphorquinone (Ambeed, 98%), N‐Phenylglycine (Ambeed, 98%), Acrylic acid (Thermo Scientific Chemicals, 99.5%), Polyethylene glycol (Thermo Scientific Chemicals, M_n_ 1000), Acryloyl chloride (Sigma–Aldrich, 97%), Hydroquinone monomethyl ether (Ambeed, 98%), 2‐[2‐[(Tert‐butoxycarbonyl)amino]ethoxy]ethanol (Ambeed, 98%), Trifluoroacetic acid (Sigma–Aldrich, 99%), Cyclohexyl isothiocyanate (Thermo Scientific Chemicals, 98%), Triethylamine (Fisher, 99%), Dichloromethane (Fisher, anhydrous), Hexane (Fisher, 99%), Ethyl acetate (Fisher, 99%), Tetrahydrofuran (Fisher, 99%), Diethyl ether (Fisher, 99%), methanol (Fisher, 99%).

### Instruments and Characterization Methods

^1^H and ^13^C NMR spectra were recorded on a Bruker Avance III 400 MHz NMR spectrometer and Bruker Avance II 500 MHz NMR spectrometer. Chemical shifts were referenced to the residual solvent peak in CDCl_3_, DMSO‐*d*_6,_ ACN‐*d*_3_, and D_2_O at δ 7.26 ppm, 2.50 ppm, 1.94 ppm, and δ 4.79 ppm for ^1^H NMR, respectively. Additionally, ^13^C NMR solvent signals of DMSO‐*d*_6_ and CDCl_3_ were 39.52 and 77.16 ppm.^[^ ^27^ ^]^

Liquid chromatography‐mass spectrometry (LC‐MS) analysis was performed using a Shimadzu LC‐2060C 3D liquid chromatograph coupled with a Shimadzu‐2020 mass spectrometer (m/z range: 100–2000 Da). Separation was conducted on a C18 HPLC column maintained at 40 °C. The mobile phases consisted of water (0.1% formic acid) and acetonitrile (0.1% formic acid), with a constant elution of acetonitrile:H_2_O (70:30, v/v) over 10 min at a flow rate of 0.40 mL·min^−1^.

Gel permeation chromatography (GPC) was performed in N,N‐dimethylformamide with 0.1 wt.% LiBr on a Nexera GPC System (Shimadzu Inc.) equipped with refractive index detectors and two columns (GPC‐KD‐803 and GPC‐KD‐805, 8 mm × 300 mm). Polyethylene glycol standards (Agilent, 238–44000 Da.) were used for calibration.

Differential Scanning Calorimetry (DSC) Analysis was performed using a differential scanning calorimeter (TA Instruments DSC250) calibrated with (standard indium). Hydrogel samples were first lyophilized and further dried in a vacuum oven at 100 °C for 24 h to remove residual moisture. Dried hydrogel samples (5–10 mg) were accurately weighed and sealed in aluminum pans; an empty pan was used as the reference. The samples were first heated to 160 °C and held isothermally for 3 min to eliminate thermal history, then cooled to −30 °C at a rate of 10 °C min^−1^ and held at −30 °C for 3 min. The samples were subsequently heated to 160 °C at the same rate. Data from the second heating cycle were used to determine the glass transition temperature.

ATR‐FTIR spectra were recorded using an FTIR spectrometer (Thermo Scientific Nicolet iS50) equipped with a (diamond, Smart iTX) attenuated total reflectance (ATR) accessory. The hydrogel precursor sample was applied as a droplet on the crystal surface with a water background.

The UV/vis spectrum was measured using UV/vis spectrometer (NanoDrop‐One^c^ spectrometer, Thermo Fisher Scientific Inc.). For solutions, the sample was dissolved in water at an appropriate concentration (0.66 mg mL^−1^). For the solid film test, the sample in DCM (0.66 mg mL^−1^) was drop‐casted on the quartz cuvette (20 µL). Photo‐polymerization experiments were conducted under LED light (Dental wireless curing light, maximum power of 5 W, 1400 mW cm^−2^). Photocleavage experiments were conducted using a light‐emitting diodes (LED) controlling system (Mightex, Dual‐mode universal LED controller, SLC‐MA/CAxx‐MU series) with a 365 nm LED light source (LCS‐0365‐04‐xx). Microscope images were acquired using a BZ‐X710 all‐in‐one fluorescence microscope (Keyence Inc.) in phase‐contrast mode with a 4 × objective lens (NA 0.13, WD 16.5 mm).

Photo‐degradation kinetics of ONB‐PEGDMA were assessed using ^1^H NMR spectroscopy in D_2_O, ACN or ACN/D_2_O under 365 nm irradiation at varying intensities and times. The percent of ONB‐PEGDMA remaining was determined by integrating its characteristic proton signals (δ 5.24 ppm) and normalizing it to the internal standard. Kinetic data were analyzed using a first‐order rate equation: ln(c*_t_*/c_0_) = ‐*kt*, where c*_t_* and c_0_ represent the normalized concentration at time *t* and 0, respectively, and *k* is the rate constant.

The precursor adhesive solutions were prepared by first mixing acrylic acid (30 wt.%), poly(ethylene glycol) diacrylate (PEGDA, 2 wt.%), and camphorquinone initiator (0.1 wt.%), followed by the addition of deionized water and N‐phenylglycine co‐initiator (0.1 wt.%). A volume of 20 µL of the precursor solution was applied onto a glass slide, covered with a second slide to form an overlapped area of 12 mm  ×  12 mm, and cured under 470 nm light for 3 min to achieve full crosslinking. Adhesive strength was tested on a universal tester (EZ‐LX, Shimadzu Inc.) with a crosshead speed of 1 mm min^−1^. The maximum force was recorded and divided by the adhesion area to obtain the tensile stress as the adhesive strength. For light‐triggered debonding tests, cured assemblies were irradiated with 365 nm light at varying intensities and durations, followed by immediate mechanical testing to assess adhesion loss. To test debonding time under a constant load, hydrogel glues were subjected to a sustained 10 N tensile force on the universal tester and irradiated with 365 nm light; the time until adhesive failure was recorded.

Scanning electron microscopy (SEM) analysis was performed using a Zeiss Sigma 500 VP Analytical FE‐SEM at 3 kV with Oxford Microanalysis. Hydrogel samples were flash‐frozen in liquid nitrogen for 3 min, fractured perpendicularly with tweezers, and subsequently freeze‐dried. Fractured surfaces were sputter‐coated with Pd/Au for 30 s using a Denton Desk V TSC sputter coater prior to imaging.

Rheological measurements were performed using a TA Instruments DHR‐2 rheometer at room temperature. The viscoelastic properties of the hydrogel glues were monitored in a time‐sweep under a strain of 1% and an angular frequency of 10 rad × s^−1^. The sample was deposited onto a flat glass bottom plate, and a 20 mm diameter upper parallel plate was lowered to a gap of 0.15 mm. Photopolymerization was initiated by irradiating the sample with 470 nm light for 3 min, followed by a 5‐min equilibration. Subsequent exposure to 365 nm light was used to induce photocleavage of the network.

### Synthetic Procedures—Synthesis of 2‐nitrobenzene‐1,3‐diol (ONB‐diOH)

The synthesis was performed with slight modifications based on a reported method.^[^ ^21c^ ^]^ A solution of 2‐nitroisophthalic acid (**ONB‐diCOOH**, 4.50 g, 21.3 mmol) in anhydrous THF (100 mL) was purged with dry N_2_ for 15 min and placed in an ice‐water bath. BH_3_·THF (112.5 mL, 1.00 m in THF, 112.8 mmol) was added slowly over 30 min. The resulting solution was then stirred at room temperature for 48 h in the dark. After completion, methanol (25 mL) was added slowly to quench the reaction, followed by vacuum filtration to remove the solid byproduct. The solvents in the filtrate were removed using a rotary evaporator, and the residue was dissolved in ethyl acetate (60 mL), followed by washing with saturated saline (3 × 60 mL). The organic layer was dried over anhydrous MgSO_4_ and concentrated under vacuum using a rotary evaporator. The crude product was purified by silica gel column chromatography (hexane:ethyl acetate = 1:1) to obtain a white solid product (3.85 g, 21.0 mmol, 99%). ^1^H NMR (400 MHz, DMSO‐*d*_6_) δ 7.60 – 7.52 (m, 3H), 5.47 (t, *J* = 5.6 Hz, 2H), 4.53 (d, *J* = 5.6 Hz, 4H). ^13^C NMR (101 MHz, DMSO‐*d*_6_) δ 147.36, 134.31, 130.74, 127.51, 59.21.

### Synthetic Procedures—Synthesis of PEGMA‐COOH

The conversion of a hydroxyl group to a carboxyl group was carried out following a previously reported method.^[^ ^28^ ^]^ Poly(ethylene glycol) methacrylate (**PEGMA**, average molecular weight of 360 Da, 1.80 g, 5.00 mmol) and succinic anhydride (2.50 g, 25.0 mmol) were dissolved in anhydrous dichloromethane (DCM, 100 mL). Then, 4‐dimethylaminopyridine (DMAP, 1.22 g, 10.0 mmol) was added. The reaction mixture was stirred at room temperature for 12 h. Then, the mixture was washed by HCl (1 m, 3 x 20 mL). The organic phase was separated and dried by anhydrous MgSO_4._ The solution was then concentrated and precipitated into diethyl ether to afford the product as a white solid (2.07 g, 84%). *M*_n(NMR)_ = 494 Da. ^1^H NMR (400 MHz, CDCl_3_) δ 6.07 (s, 1H), 5.52 (s, 1H), 4.30 – 4.19 (m, 4H), 3.71 – 3.59 (m, 24H), 2.60 (s, 4H), 1.89 (s, 3H). ^13^C NMR (101 MHz, CDCl_3_) δ 176.46 – 175.75, 172.13, 167.38, 136.12, 125.80, 70.66, 70.60, 70.52, 69.11, 68.98, 63.88, 63.85, 53.52, 29.12, 28.90, 18.31.

### Synthetic Procedures—Synthesis of ONB‐PEGDMA

To a flask, PEGMA‐COOH (1.38 g, 3.00 mmol), ONB‐diOH (180 mg, 1.00 mmol), and 4‐dimethylaminopyridine (DMAP, 50 mg, 0.40 mmol) were dissolved in DCM (20 mL). The solution was cooled to 0 °C, followed by slow addition of EDC‐HCl (570 mg, 3.00 mmol). Then the reaction warmed up to room temperature and stirred for 24 h. Then, the mixture solution was washed with saturated sodium bicarbonate solution (3 × 10 mL), and the organic phase was separated and dried by anhydrous MgSO_4._ The organic phase was then evaporated, and the residue was purified by silica column chromatography (DCM:MeOH = 20:1) to afford the product as a viscous liquid (ONB‐PEGDMA, 0.90 g, 86% yield, *M*_n(GPC)_ = 0.8 × 10^3^ Da. and *Ɖ =* 1.08 from GPC, *M*_n(NMR) =_ 1.05 × 10^3^ Da. ^1^H NMR (400 MHz, CDCl_3_) δ 7.55 – 7.48 (m, 3H), 6.12 (s, 2H), 5.59 – 5.56 (m, 2H), 5.23 (s, 4H), 4.33 – 4.22 (m, 8H), 3.75 – 3.64 (m, 42H), 2.66 (s, 8H), 1.94 (s, 6H). ^13^C NMR (101 MHz, CDCl_3_) δ 172.15, 171.69, 167.44, 148.74, 136.26, 131.36, 129.96, 129.47, 125.82, 70.74, 70.71, 70.67, 69.23, 69.11, 64.02, 63.98, 62.35, 28.92, 28.88, 18.41.

### Synthetic Procedures—Synthesis of PEGDA

PEG (*M*_n_ 1000, 2.00 g, 2.00 mmol) and K_2_CO_3_ (0.83 g, 6.0 mmol) were mixed in DCM (20 mL) and cooled to 0 °C. Acryloyl chloride (0.54 g, 6.0 mmol) was added dropwise into the mixture. The reaction was allowed to warm to room temperature and stirred for 12 h. Upon completion, the mixture was washed with brine three times. The organic layer was separated, dried by anhydrous MgSO_4_, and concentrated, followed by precipitation into cold diethyl ether to afford product as a white solid (1.30 g, 59% yield, *M*_n(GPC)_ = 0.74 × 10^3^ Da. and *Ɖ =* 1.14 from GPC, *M*_n(NMR) =_ 1.095 × 10^3^ Da.). ^1^H NMR (400 MHz, CDCl_3_) δ 6.43 (dd, *J* = 17.4, 1.5 Hz, 2H), 6.15 (dd, *J* = 17.3, 10.4 Hz, 2H), 5.84 (dd, *J* = 10.4, 1.5 Hz, 2H), 4.33 – 4.30 (m, 4H), 3.75 – 3.73 (m, 4H), 3.66 – 3.64 (80H). ^13^C NMR (101 MHz, CDCl_3_) δ 166.16, 131.00, 128.35, 70.67, 70.61, 69.15, 63.72.

## Conflict of Interest

The authors declare no conflict of interest.

## Supporting information



Supporting Information

## Data Availability

The data that support the findings of this study are available in the supplementary material of this article.
