# Supplementary material for: Dual‐Wavelength Responsive Hydrogel Glue with Visible‐Light Bonding and UV‐Triggered Debonding via Ortho‐Nitrobenzyl Cleavage
Source: Adv Sci (Weinh). 2025 Aug 4;12(40):e07809. doi: 10.1002/advs.202507809 (PMC12561338; doi:10.1002/advs.202507809)
Supplement: Supplementary file 1 — Supporting Information [file ADVS-12-e07809-s001.docx]

SUPPORTING INFORMATION

**Dual-Wavelength Responsive Hydrogel Glue With Visible-Light Bonding And UV-Triggered Debonding Via *Ortho*-Nitrobenzyl Cleavage**

Huaming Wang^1^, Xianyan Shen^1^, Changling Du^1^, Xian-You Liu^1^, Anyu Yang^1^, Yanni Cao^1^, Aijie Han^2^, Qihan Liu^3^, Jennifer Laaser^2^, Wei Zhang^1,2 *^

^1^Department of Pharmaceutical Science, University of Pittsburgh, Pittsburgh, PA, USA

^2^Department of Chemistry, University of Pittsburgh, Pittsburgh, PA, USA

^3^Department of Mechanical Engineering, University of Pittsburgh, Pittsburgh, PA, USA

*Corresponding authors, email: [weizhang@pitt.edu](mailto:weizhang@pitt.edu)

**ADDITIONAL FIGURES**


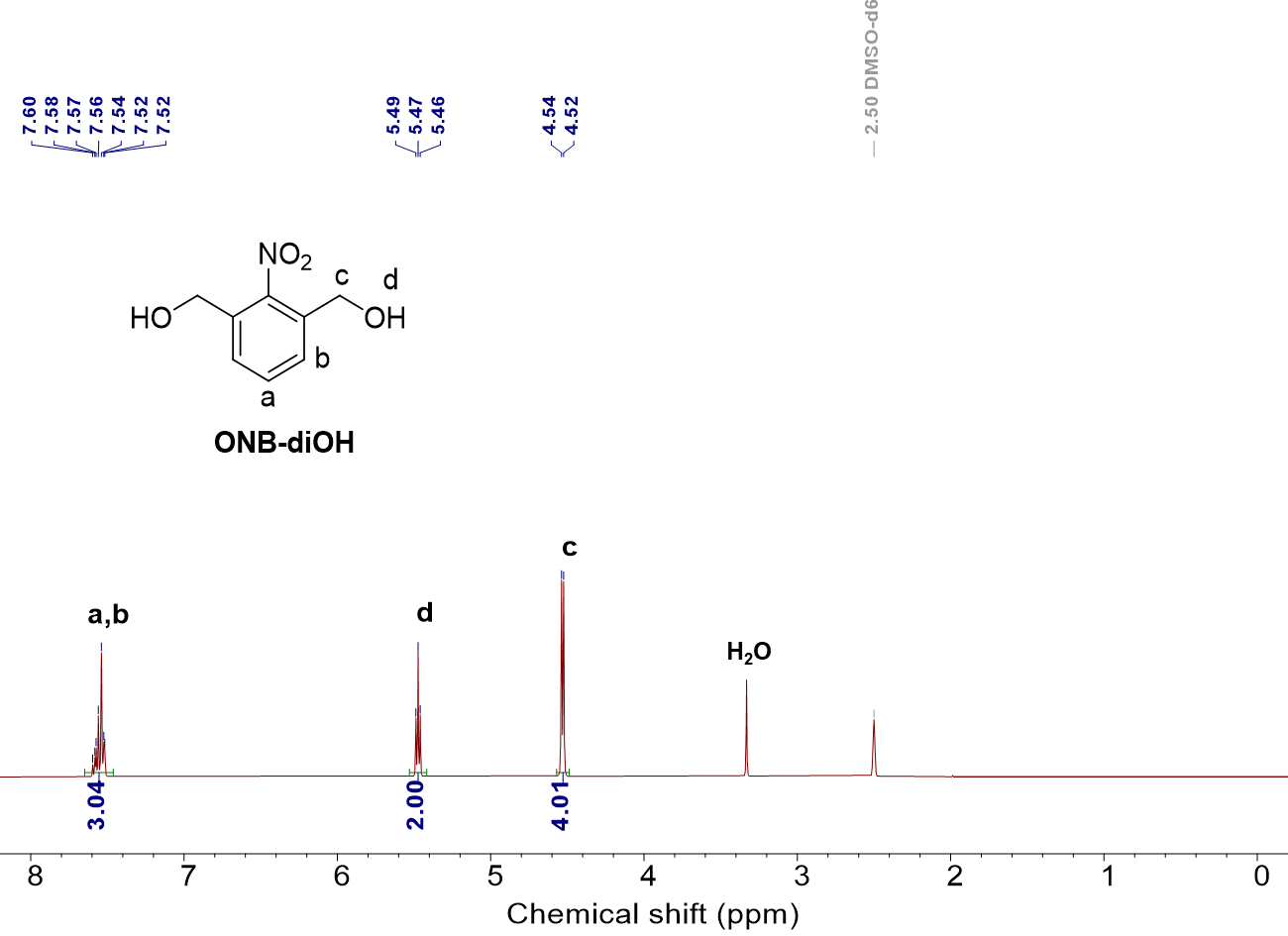


Figure S1. ^1^H NMR spectrum of **ONB-diOH.**


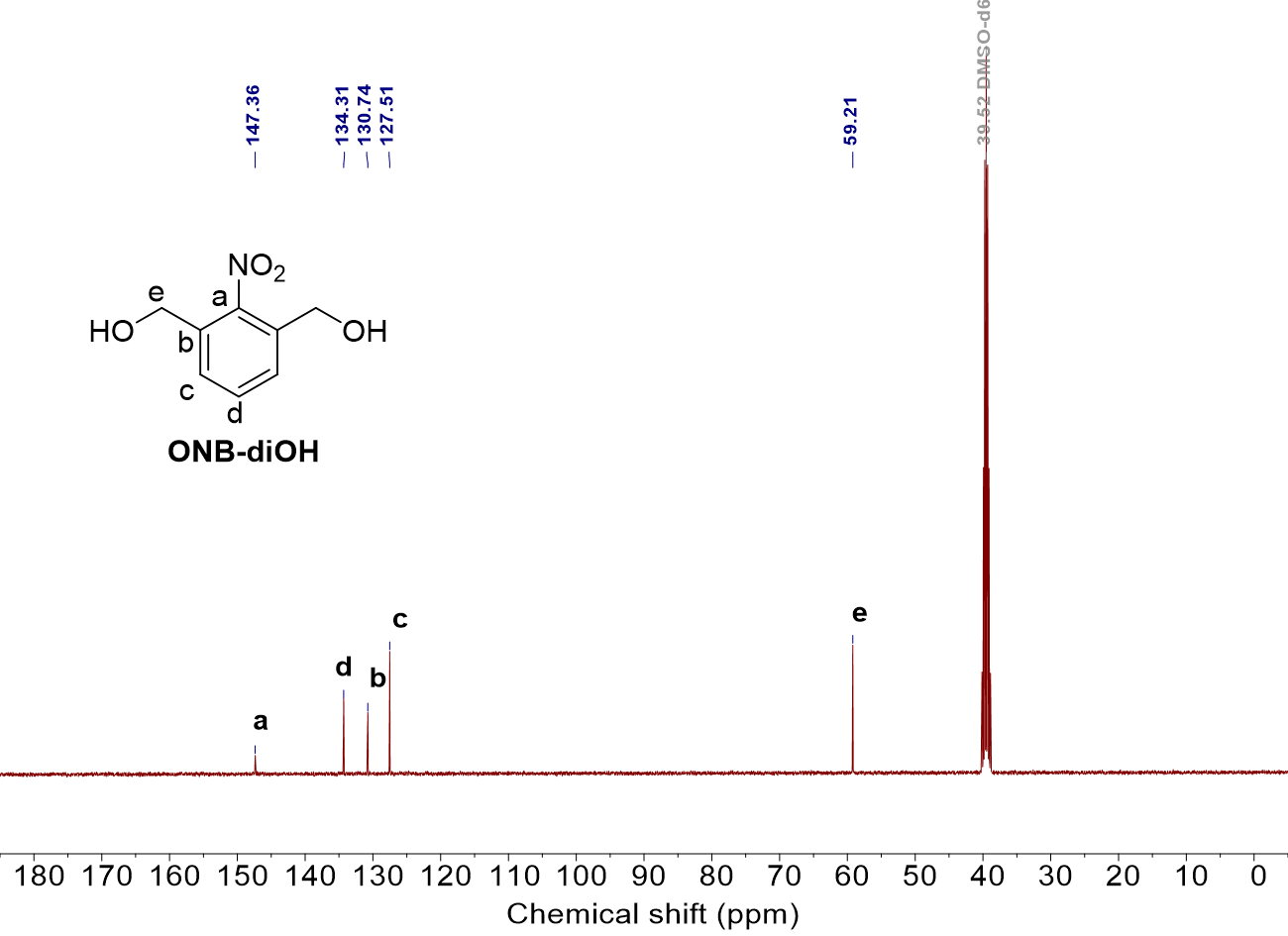


Figure S2. ^13^C NMR spectrum of **ONB-diOH.**


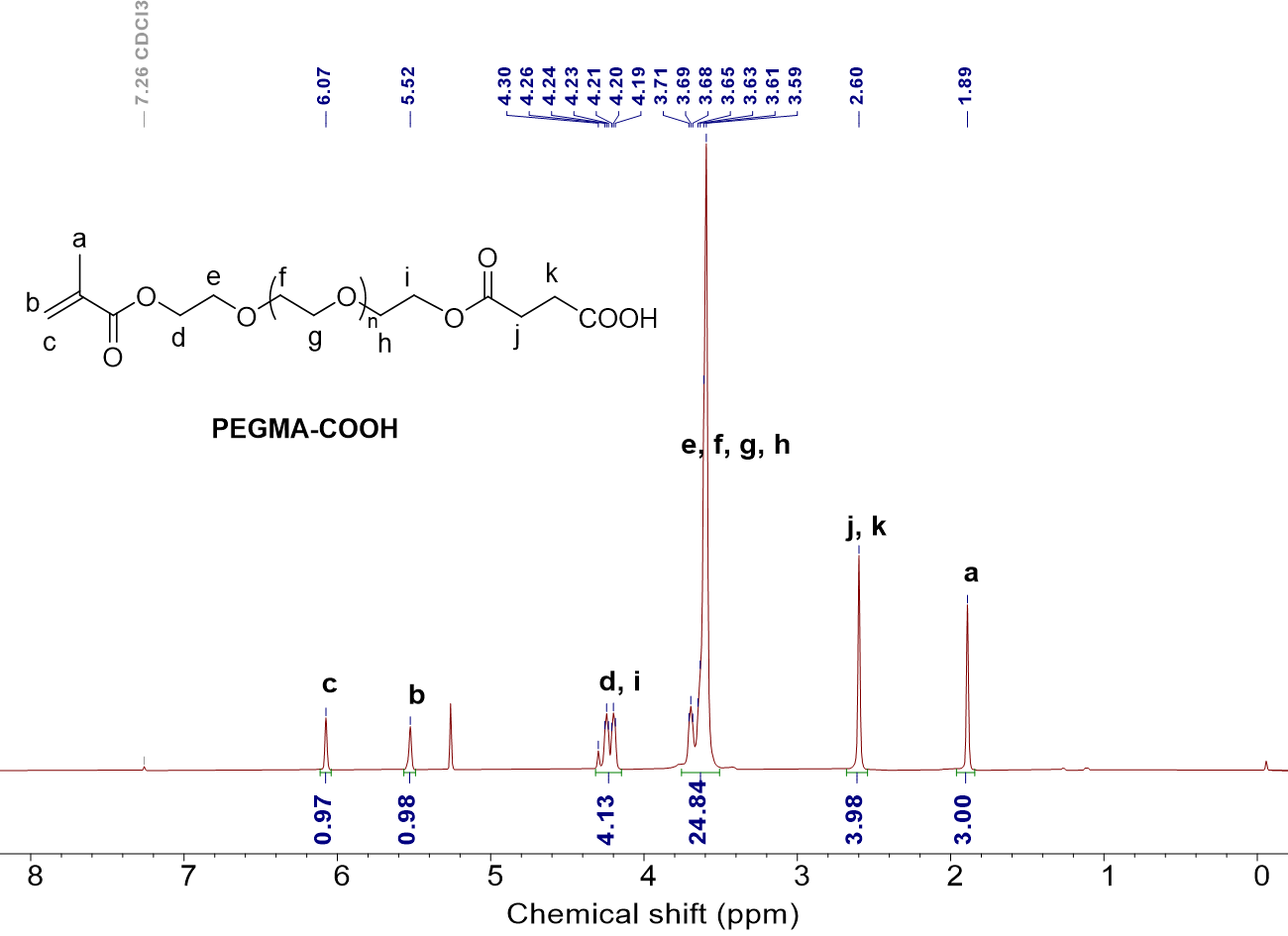


Figure S3. ^1^H NMR spectrum of **PEGMA-COOH.**


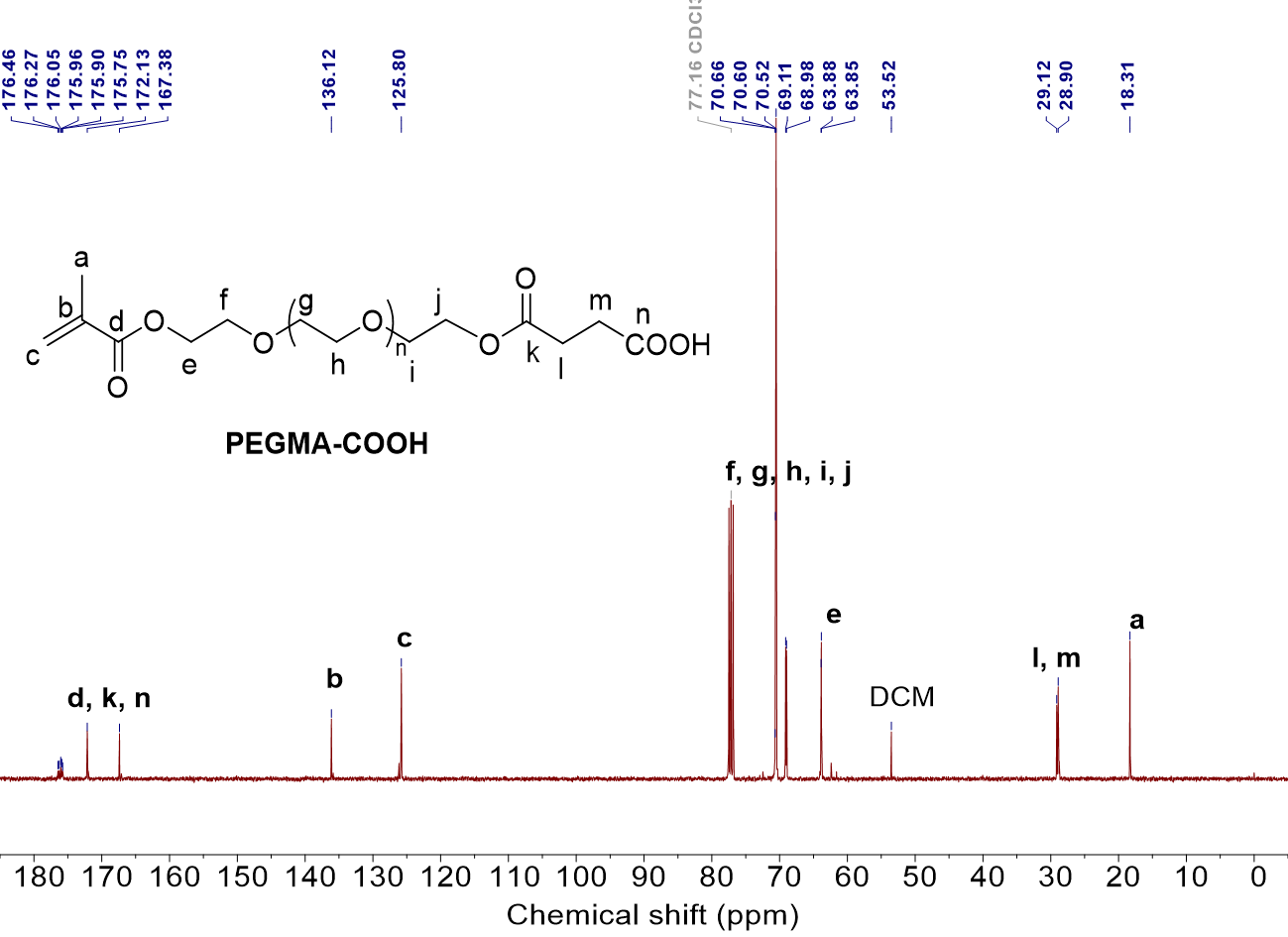


Figure S4. ^13^C NMR spectrum of **PEGMA-COOH.**


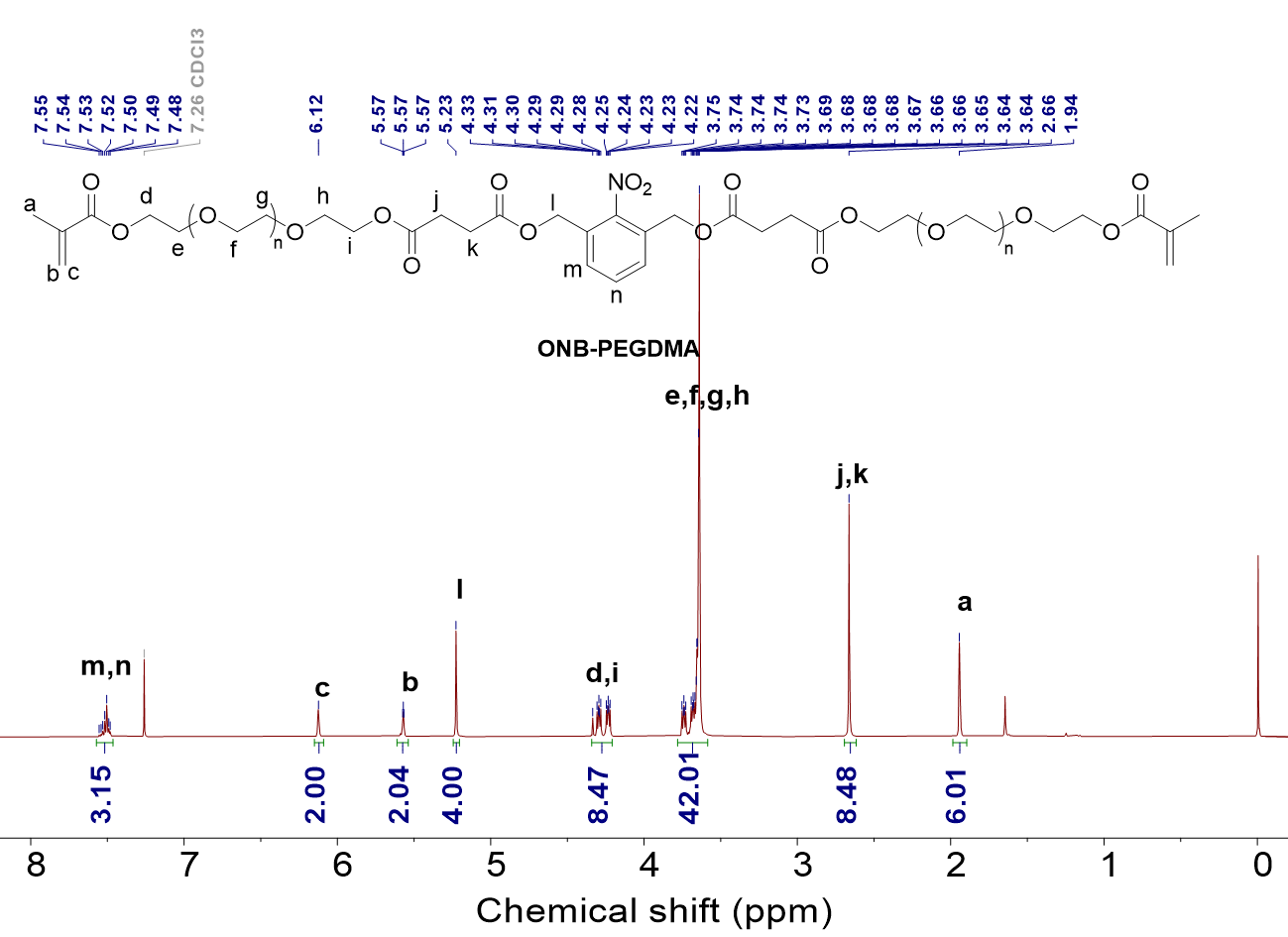


Figure S5. ^1^H NMR spectrum of **ONB-PEGDMA.**


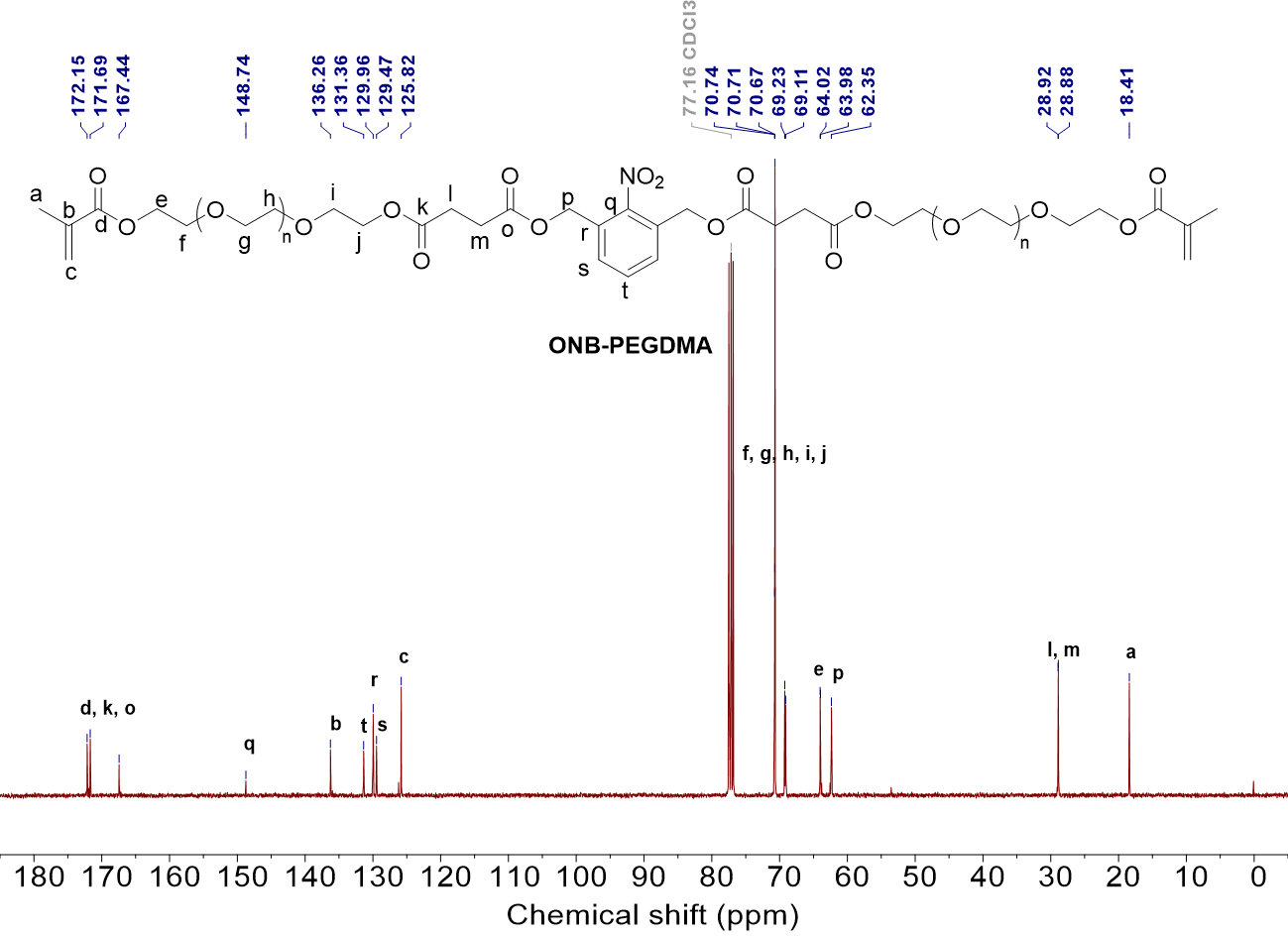


Figure S6. ^13^C NMR spectrum of **ONB-PEGDMA.**

**
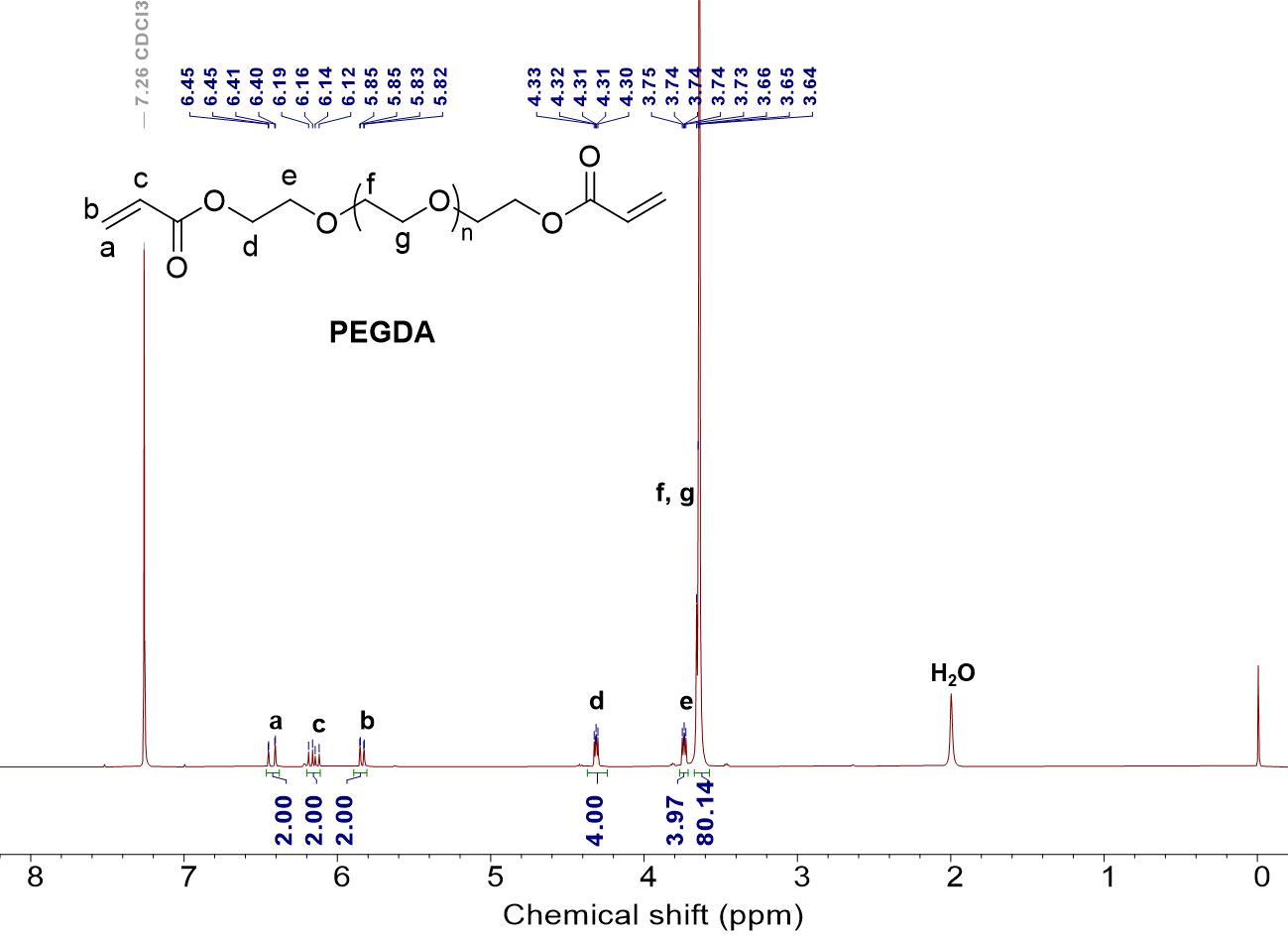
**

Figure S7. ^1^H NMR spectrum of **PEGDA.**

**
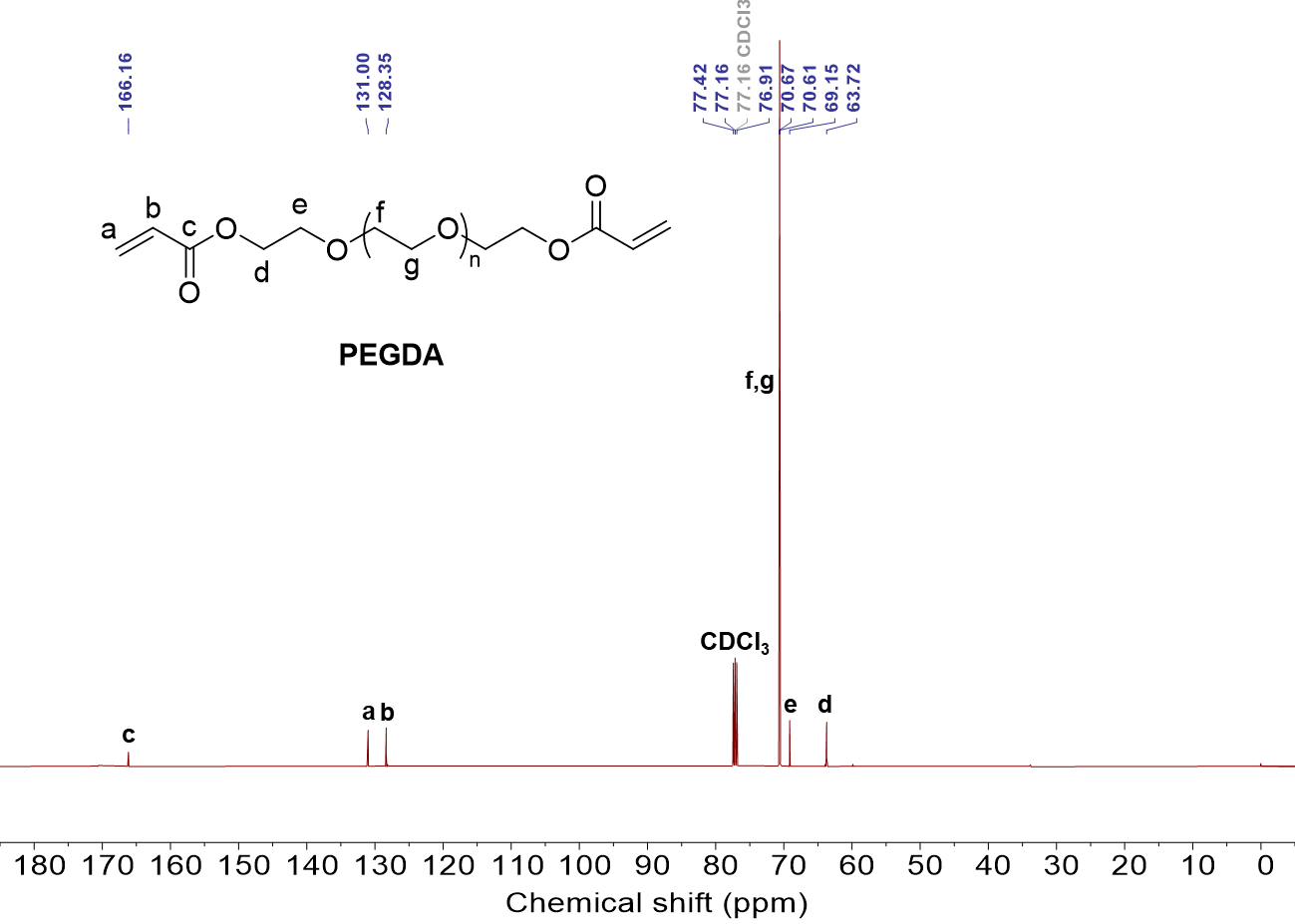
**

Figure S8. ^13^C NMR spectrum of **PEGDA.**

Figure S9. GPC curves of **ONB-PEGDMA and PEGDA.**


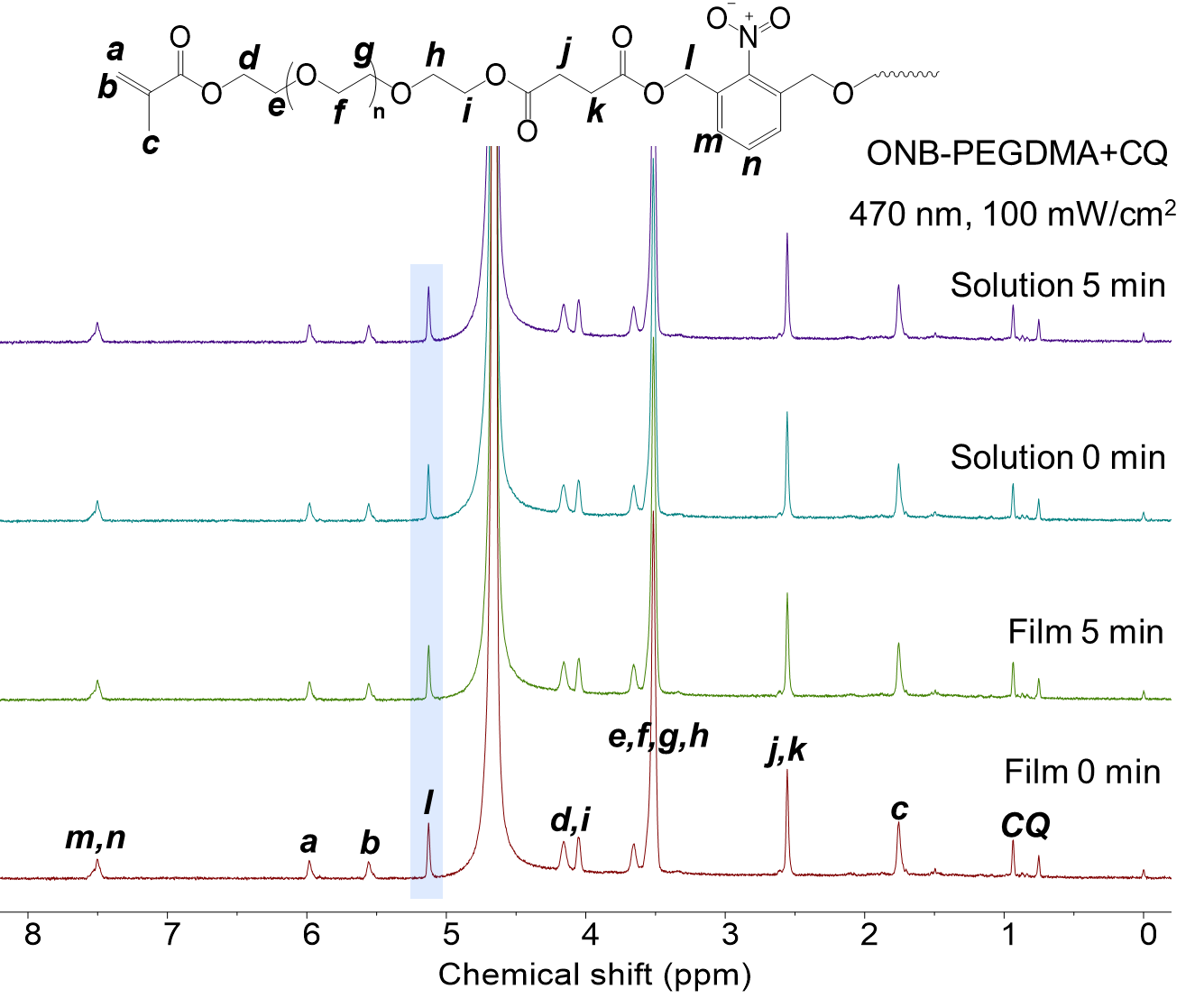


Figure S10. ^1^H NMR spectra of ONB-PEGDMA + CQ (20:1) under 470 nm irradiation in D_2_O.


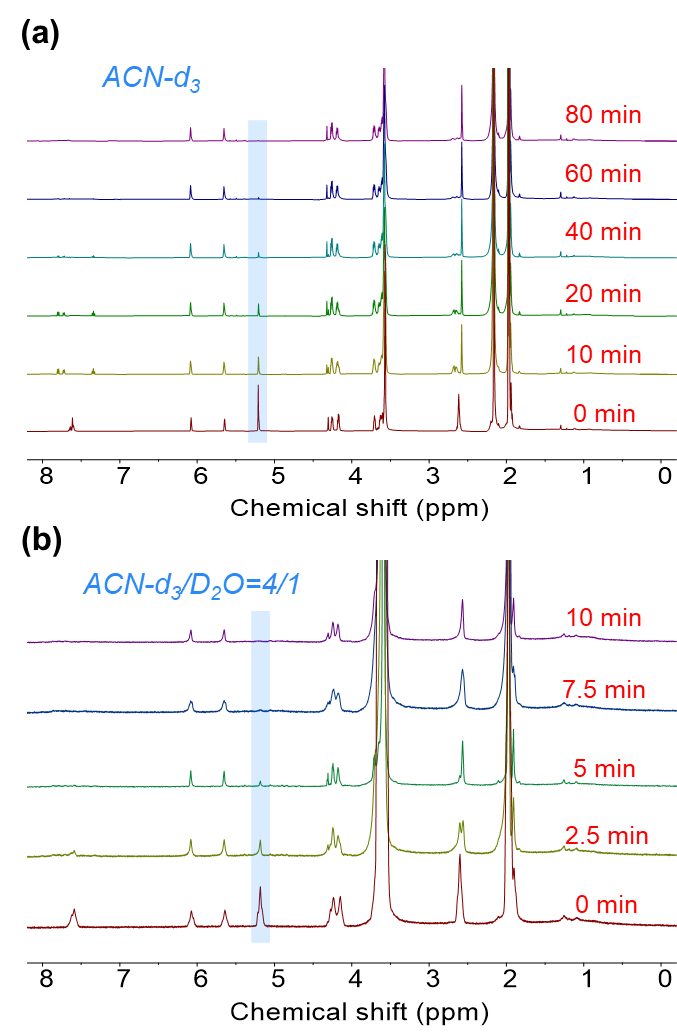


Figure S11. ^1^H NMR spectra of ONB-PEGDMA (c=0.66 mg/mL) under 365 nm irradiation (a) in ACN-*d*_3_ and (b) ACN-*d*_3_/D_2_O (4/1).


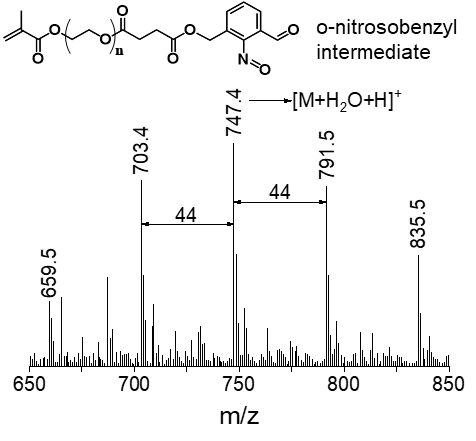


Figure S12. MS spectrum of the o‑nitrosobenzyl intermediate from ONB-PEGDMA photodegradation (100 mW/cm^2^, 1 min).


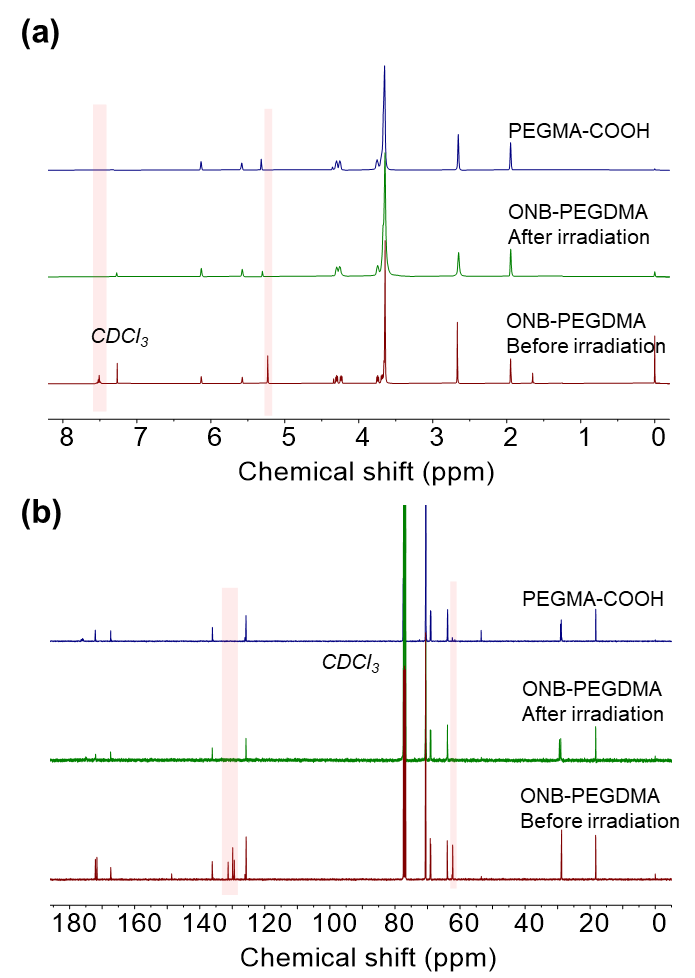


Figure S13. (a)^1^H NMR and (b) ^13^C NMR spectra of PEGMA-COOH and ONB-PEGDMA before and after irradiation. ONB-PEGDMA was dissolved in H_2_O and irradiated under 365 nm with 100 mW/cm^2^ for 10 min. Then, the mixture was freeze-dried and dissolved in CDCl_3_ for NMR test.


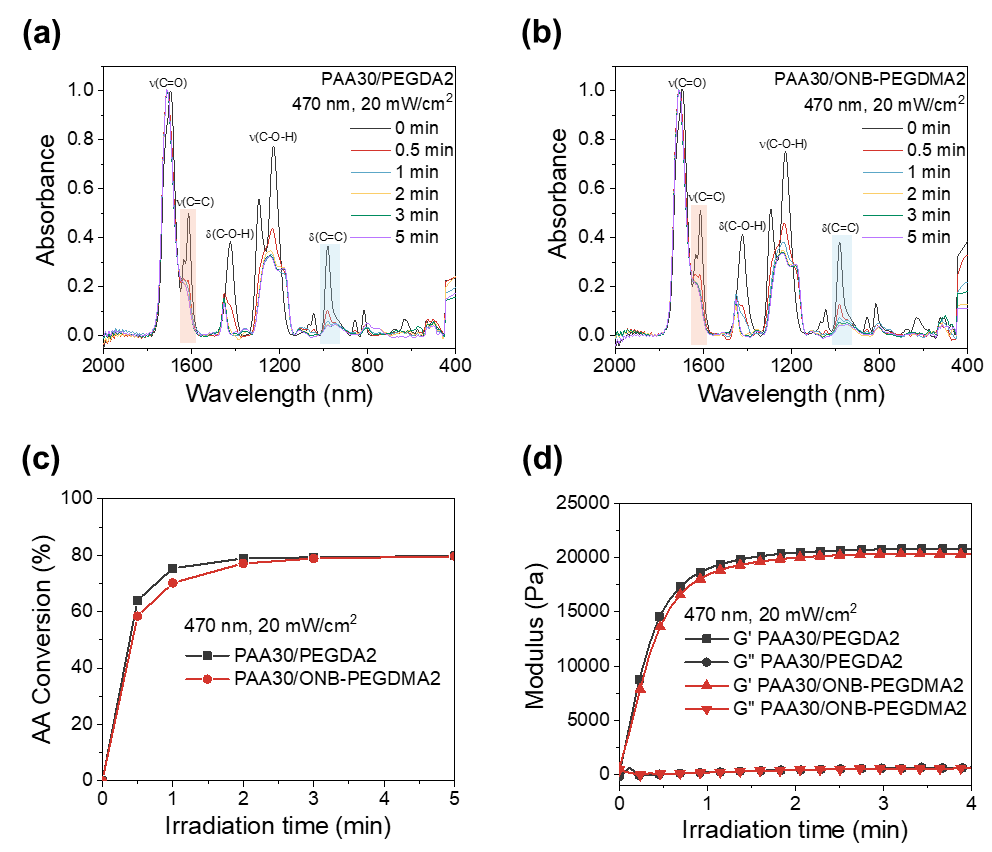


Figure S14. Gelation kinetics of the hydrogel. (a) FTIR spectra of PAA30/PEGDA2 and (b) PAA30/ONB-PEGDMA2 precursor after different duration of 470 nm irradiation. (c) Acrylic acid conversion calculated from the decrease of δ (C=C) absorption band in the FTIR spectra. (d) In situ rheological measurements of PAA30/PEGDA2 and PAA30/ONB-PEGDMA2 under 470 nm irradiation


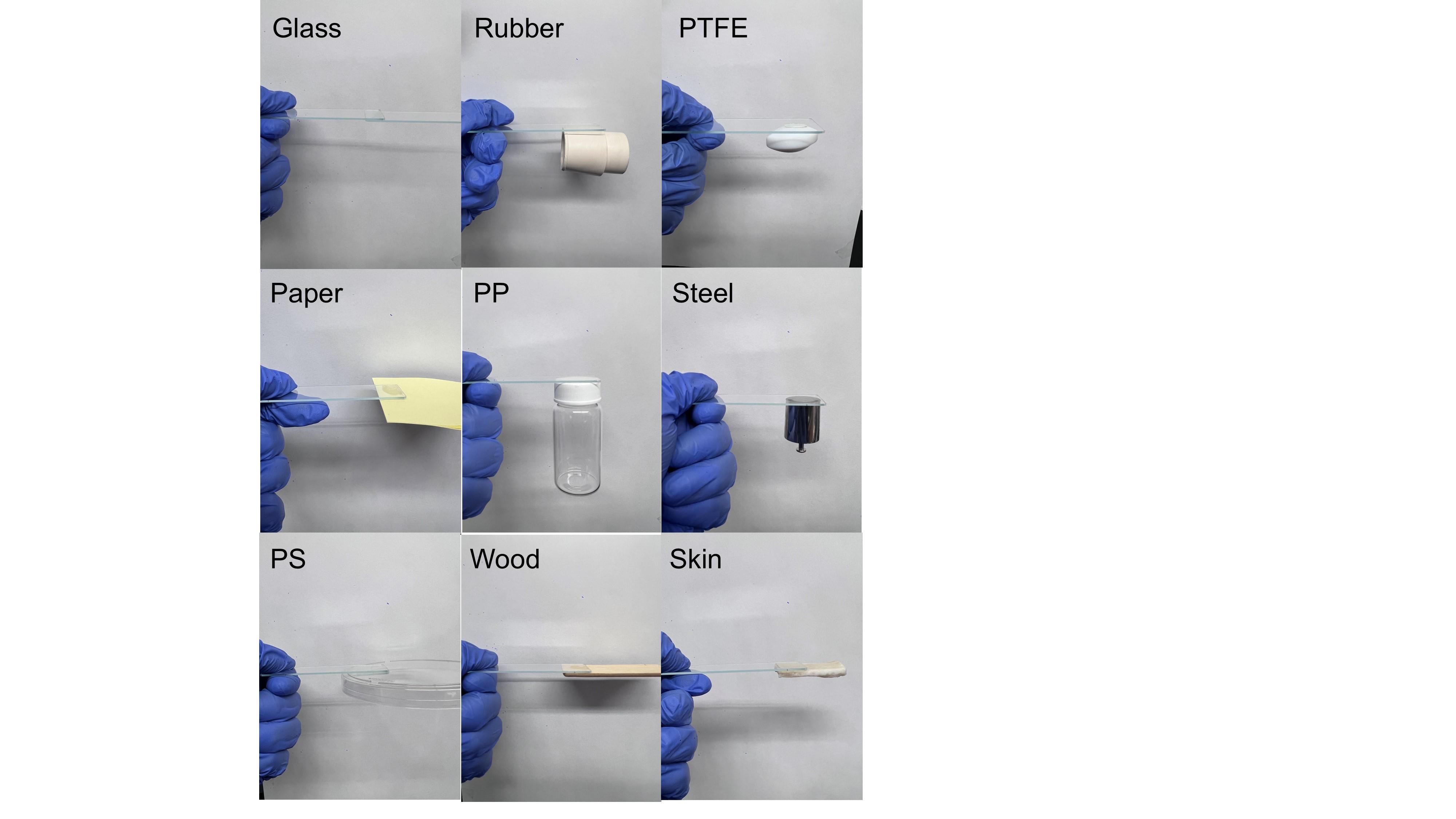


Figure S15. Photographs of PAA30-ONB-PEGDMA adhered to various materials.

Figure S16. Stress-displacement curves of PAA30/ONB-PEGDMA2 after different irradiation time under 365 nm light at 100 mW/cm^2^.

Figure S17. Second heating curves from DSC analysis of freeze-dried samples. Shown are PAA and PAA30/ONB-PEGDMA hydrogels containing 1%, 2%, and 3% ONB-PEGDMA, before and after 365 nm irradiation. *T*_g_ values were extracted from the midpoint of the glass transition region.


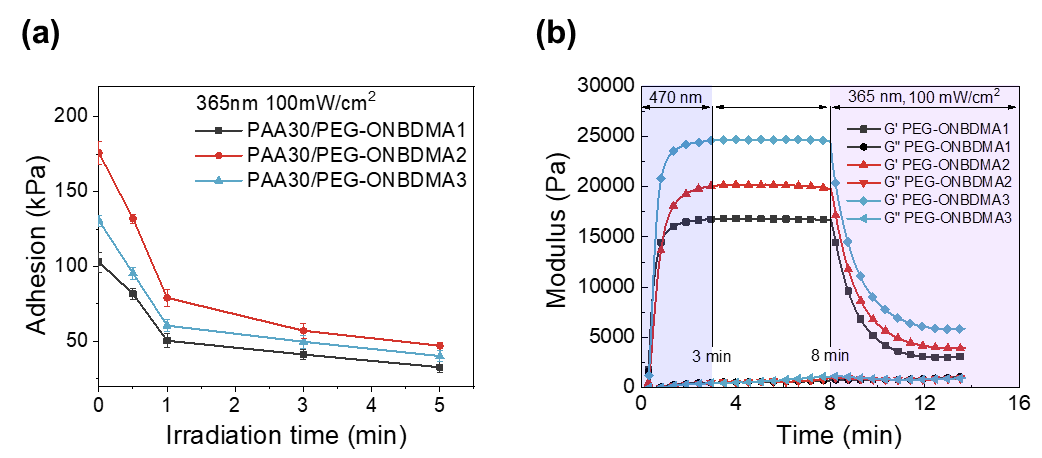


Figure S18. Photoinduced debonding of PAA30/ONB-PEGDMA hydrogels with varying crosslinker content (1%, 2%, 3%). (a) Adhesion strength of PAA30/ONB-PEGDMA from lap shear tests after different irradiation times and intensities. (b) Rheological analysis showing moduli changes of PAA30/ONB-PEGDMA during hydrogel formation under 470 nm light (20 mW/cm^2^), followed by photoinduced decrosslinking under 365 nm light (100 mW/cm^2^)


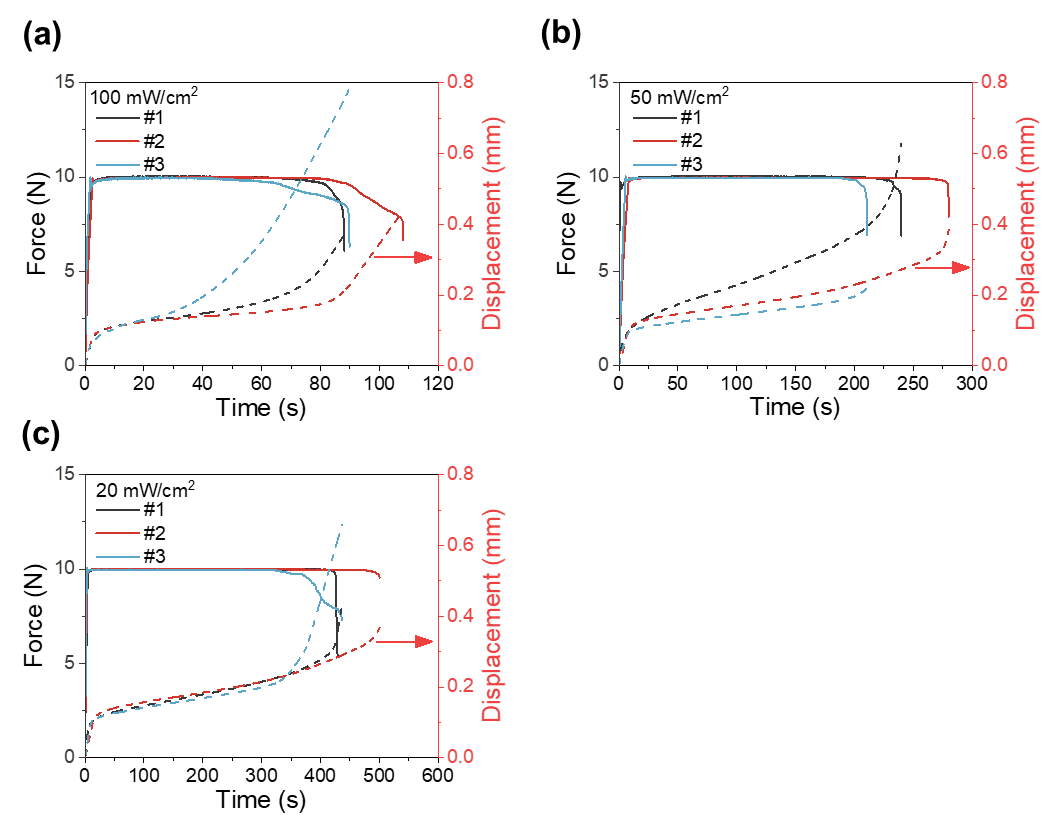


Figure S19. Force–time curves of the adhesive debonding process under a constant tensile load of 10 N. Solid lines (left y-axis) represent force; dashed lines (right y-axis) represent displacement during the test. Samples were irradiated at different light intensities (a) 100 mW/cm^2^, (b) 50 mW/cm^2^, and (c) 20 mW/cm^2^.
